# Regulation of *Neurod1* Contributes to the Lineage Potential of Neurogenin3+ Endocrine Precursor Cells in the Pancreas

**DOI:** 10.1371/journal.pgen.1003278

**Published:** 2013-02-07

**Authors:** Teresa L. Mastracci, Keith R. Anderson, James B. Papizan, Lori Sussel

**Affiliations:** 1 Department of Genetics and Development, Russ Berrie Medical Pavilion, Columbia University, New York, New York, United States of America; 2 Molecular Biology Program, University of Colorado Denver Health Sciences Center, Aurora, Colorado, United States of America; Vanderbilt University Medical Center, United States of America

## Abstract

During pancreatic development, transcription factor cascades gradually commit precursor populations to the different endocrine cell fate pathways. Although mutational analyses have defined the functions of many individual pancreatic transcription factors, the integrative transcription factor networks required to regulate lineage specification, as well as their sites of action, are poorly understood. In this study, we investigated where and how the transcription factors Nkx2.2 and Neurod1 genetically interact to differentially regulate endocrine cell specification. In an *Nkx2.2* null background, we conditionally deleted *Neurod1* in the Pdx1+ pancreatic progenitor cells, the Neurog3+ endocrine progenitor cells, or the glucagon+ alpha cells. These studies determined that, in the absence of Nkx2.2 activity, removal of *Neurod1* from the Pdx1+ or Neurog3+ progenitor populations is sufficient to reestablish the specification of the PP and epsilon cell lineages. Alternatively, in the absence of Nkx2.2, removal of *Neurod1* from the Pdx1+ pancreatic progenitor population, but not the Neurog3+ endocrine progenitor cells, restores alpha cell specification. Subsequent *in vitro* reporter assays demonstrated that Nkx2.2 represses *Neurod1* in alpha cells. Based on these findings, we conclude that, although Nkx2.2 and Neurod1 are both necessary to promote beta cell differentiation, Nkx2.2 must repress *Neurod1* in a Pdx1+ pancreatic progenitor population to appropriately commit a subset of Neurog3+ endocrine progenitor cells to the alpha cell lineage. These results are consistent with the proposed idea that Neurog3+ endocrine progenitor cells represent a heterogeneous population of unipotent cells, each restricted to a particular endocrine lineage.

## Author Summary

Diabetes mellitus is a family of metabolic diseases that can result from either destruction or dysfunction of the insulin-producing beta cells of the pancreas. Recent studies have provided hope that generating insulin-producing cells from alternative cell sources may be a possible treatment for diabetes; this includes the observation that pancreatic glucagon-expressing alpha cells can be converted into beta cells under certain physiological or genetic conditions. Our study focuses on two essential beta cell regulatory factors, Nkx2.2 and Neurod1, and demonstrates how their genetic interactions can promote the development of other hormone-expressing cell types, including alpha cells. We determined that, while Nkx2.2 is required to activate *Neurod1* to promote beta cell formation, Nkx2.2 must prevent expression of *Neurod1* to allow alpha cell formation. Furthermore, the inactivation of *Neurod1* must occur in the earliest pancreatic progenitors, at a stage in the differentiation process earlier than previously believed. These studies contribute to our understanding of the overlapping gene regulatory networks that specify islet cell types and identify the importance of timing and cellular context for these regulatory interactions. Furthermore, our data have broad implications regarding the manipulation of alpha cells or human pluripotent stem cells to generate insulin-producing beta cells for therapeutic purposes.

## Introduction

The destruction or dysfunction of the insulin-producing beta cells of the pancreas contributes to a family of metabolic diseases known as diabetes mellitus. Given that the specification of the three major cell types in the pancreas, endocrine, exocrine and ductal cells, occurs in the embryo, understanding the normal course of pancreas development will ultimately facilitate the generation of insulin-producing beta cells from alternative cell sources for beta cell replacement therapies [1], [2], [3]. Single knockout mouse models have determined the relative importance of many transcription factors in the process of endocrine cell specification and differentiation. Of particular significance, deletion of the basic helix-loop-helix transcription factor *Neurogenin3* (*Neurog3*; *Ngn3*) results in the loss of the hormone-producing cell types [4]. Subsequent lineage tracing experiments confirm that hormone-expressing endocrine cell types, including alpha cells (expressing glucagon), beta cells (insulin), delta cells (somatostatin), epsilon cells (ghrelin), and PP cells (pancreatic polypeptide), are Neurog3-derived [5], [6].

A recent study suggested that each Neurog3+ endocrine progenitor cell within the population is destined to become a single hormone+ cell type [7]. The idea that endocrine progenitor cells are unipotent implies that the transcription factor code responsible for the differentiation of each hormone+ cell type may be delineated before endocrine progenitors are specified. In support of this hypothesis, forced expression of factors within the Pdx1+ pancreatic progenitor cells can affect the resulting complement of differentiated endocrine cells [8], [9], [10]. Ultimately, the proper timing and location of transcription factor expression and function during pancreas development is essential for the appropriate differentiation of all the hormone-expressing endocrine cells.

The homeobox transcription factor Nkx2.2 is a particularly interesting pancreatic regulatory protein due to its dynamic expression pattern and cell-specific regulatory activities. Nkx2.2 is widely expressed throughout the early undifferentiated pancreatic epithelium, but gradually becomes restricted to beta cells and a large subset of alpha and PP cells [11], [12]. Despite its early and widespread expression, deletion of Nkx2.2 specifically affects later endocrine lineage specification: beta cells do not form, alpha and PP cell numbers are decreased, and there is a significant increase in the ghrelin cell population. Furthermore, while Nkx2.2 is expressed in both glucagon+ alpha cells and insulin+ beta cells [13] and the physical interaction of Nkx2.2 with the co-repressor Groucho3 (Grg3; Tle3) occurs in both cell types, the recruitment of a repressor complex to the promoter of the homeobox transcription factor *Arx* occurs in beta, but not alpha cells [14], presumably due to cell-specific and/or promoter-specific protein interactions. Disruption of the Nkx2.2/Grg3 interaction results in the mis-specification of islet cell types and the subsequent trans-differentiation of beta cells into alpha cells [14]. Studies of other developmental systems, including muscle and CNS, have also provided examples of how a single transcription factor can differentially regulate cell specification [15], [16], [17], [18]. Altogether these studies demonstrate that cell-specific transcription factor regulation plays a fundamental role in cell fate determination and the maintenance of cell identity.

While single knockout mouse models can uncover the role of a specific factor in the process of cell fate determination [19], [20], [21], compound deletion mutants demonstrate how multiple transcription factors work together to permit or restrict the differentiation of specific lineages. Whereas the deletion of *Arx* results in the loss of alpha cells and an increase in beta and delta cells [19], [22], deletion of *Nkx2.2* affects all islet cell types in the pancreas except the delta cell population [12]. Interestingly, simultaneous deletion of these two factors revealed for the first time that Nkx2.2 was required to repress *somatostatin* in the ghrelin-expressing epsilon cell lineage [23], [24]. Furthermore, the simultaneous deletion of *Nkx2.2* and the beta cell transcription factor *Neurod1* identified an unexpected epistatic relationship between these factors that regulates the formation of the non-beta cell types [25]. While deletion of *Neurod1* does not affect the formation of alpha or beta cells, alpha cells are reduced late in development and beta cells undergo catastrophic apoptosis by birth [26]. In contrast, the null mutation of *Nkx2.2* results in a severe reduction in alpha cells, and beta cells are completely absent [12], [27]. Despite the expression of Nkx2.2 and Neurod1 in beta cells [13], [26], [28] and the severe phenotypes associated with beta cells in both single knockout mice [12], [26], the simultaneous deletion of *Neurod1* and *Nkx2.2* did not alter the beta cell phenotype but rather restored alpha cell and PP cell formation, while simultaneously reducing the ghrelin-expressing epsilon cells, which are over abundant in the *Nkx2.2* null pancreas [25]. These examples demonstrate that deciphering the complex pancreatic gene regulatory network will provide valuable insight into the cellular processes required to generate each islet cell type, and will facilitate the *in vitro* differentiation of functional insulin-producing cells for therapeutic purposes.

The *Nkx2.2^−/−^;Neurod1^−/−^* (*Nkx2.2^null^;Neurod1^null^*) compound mutant provides a useful model for how two transcription factors coordinately regulate the specification of multiple endocrine cell types. Our study aimed to dissect the cooperative roles of Nkx2.2 and Neurod1, and determine specifically where and how these factors work together to permit endocrine cell formation in the pancreas. The result of this analysis demonstrated that in the absence of Nkx2.2, deletion of *Neurod1* in the Pdx1+ pancreatic progenitors resulted in restoration of the alpha, PP and epsilon cells; however, deletion of *Neurod1* from the Neurog3+ endocrine progenitor cells restored the PP and epsilon cells, but only a small population of alpha cells. Using *in vitro* reporter assays we also showed that Nkx2.2 repressed *Neurod1* in certain cellular contexts. Consistent with the idea that Neurog3+ cells are unipotent [7], we hypothesize that Nkx2.2 must repress *Neurod1* in the Pdx1+ pancreatic progenitors early in development to appropriately prime the Neurog3+ endocrine progenitor cells to become alpha cells.

## Results

### In the absence of Nkx2.2, *Neurod1* deletion in Pdx1+ pancreatic progenitors recapitulates the *Nkx2.2^null^;Neurod1^null^* double-knockout phenotype

**Figure 1:**
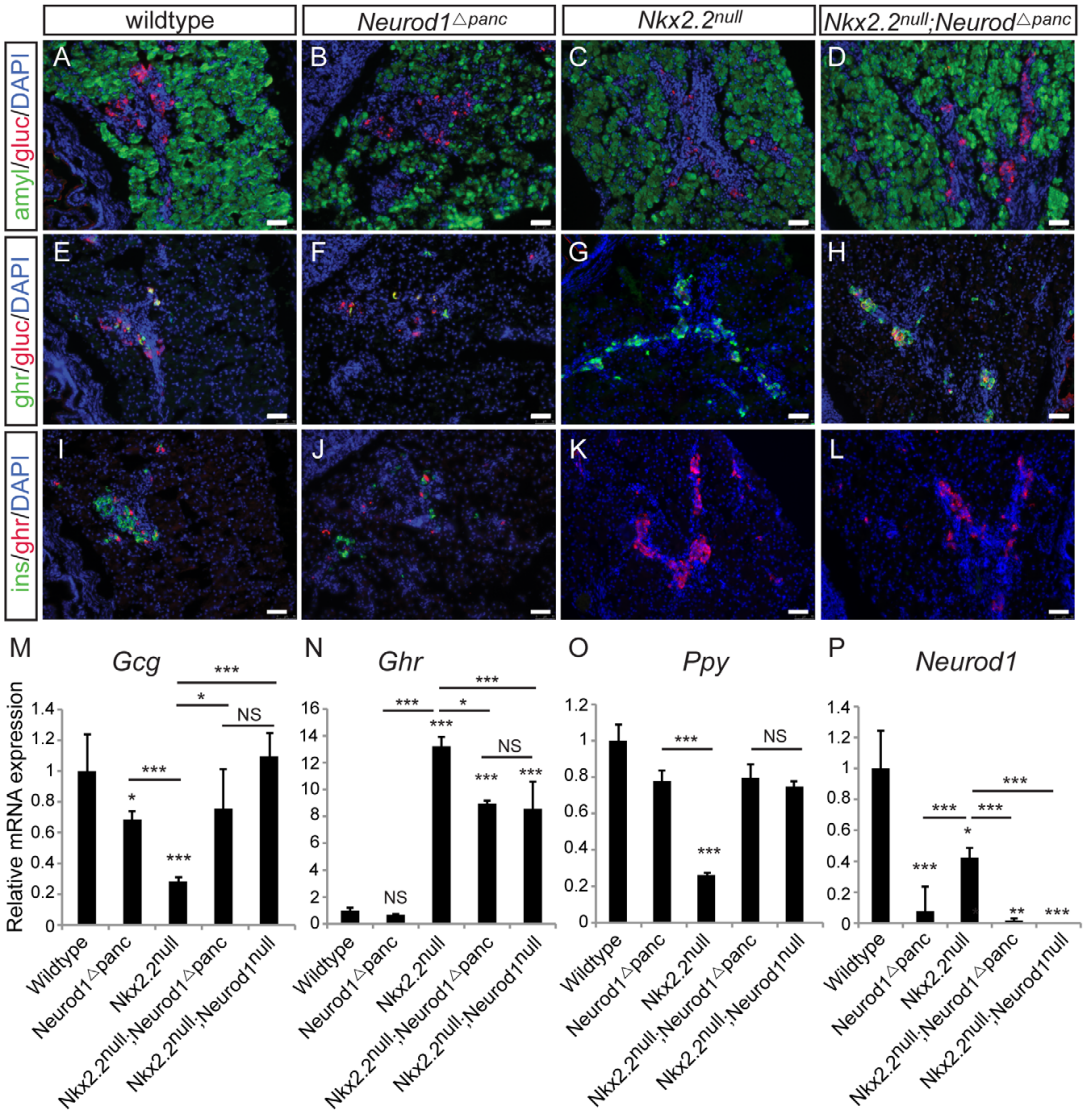
*Neurod1* deletion in the pancreas progenitors, in an *Nkx2.2* null background, phenocopies the *Nkx2.2^null^;Neurod1^null^* double knockout. Pancreatic tissue from wildtype, *Neurod1^Δpanc^*, *Nkx2.2^null^*, and *Nkx2.2^null^;Neurod1^Δpanc^* was analyzed by immunofluorescence for expression of the islet hormones glucagon (gluc), ghrelin (ghr) and insulin (ins) at P0 (A–L; white bar indicates 50 microns; DAPI marks all nuclei). Amylase (amyl) expression marks exocrine tissue in all genotypes (A–D). The quantitative expression of *glucagon* (*Gcg*) (M), *ghrelin* (*Ghr*) (N), and *pancreatic polypeptide* (*Ppy*) (O), as well as deletion of *Neurod1* (P) was determined by real time PCR using RNA extracted from wildtype, *Neurod1^Δpanc^*, *Nkx2.2^null^*, *Nkx2.2^null^;Neurod1^Δpanc^*, and *Nkx2.2^null^;Neurod1^null^* pancreas (P0; N = 3–8). Relative mRNA expression was normalized to the housekeeping gene, *cyclophilinB*. Data are represented as mean+/−SEM. * p<0.05; ** p<0.01; *** p<0.001.

To determine the precise cell type in which the genetic interaction between Nkx2.2 and Neurod1 is required for endocrine cell specification, we conditionally removed *Neurod1* from different pancreatic cell populations in the absence of Nkx2.2. We generated a pancreas-specific deletion of *Neurod1* in the *Nkx2.2* null background using *Pdx1-cre* [29] (*Nkx2.2^−/−^;Neurod1^flox/flox^;Pdx1-cre*, denoted as *Nkx2.2^null^;Neurod1^Δpanc^*). We first confirmed that the single deletion of *Neurod1* in the Pdx1+ cells (*Neurod1^Δpanc^*) phenocopied the *Neurod1^null^* mouse (Figure 1B, 1F, 1J; Figure S1), displaying the expected reduction in *insulin* and *glucagon* mRNA levels at P0 (Figure 1M; Figure S1) [26], [30]. We also demonstrated that when *Neurod1* was deleted from Pdx1+ cells in the absence of Nkx2.2, the pancreas phenotype was identical to the *Nkx2.2^null^;Neurod1^null^* mouse [25] (Figure S1). Specifically, all beta cells were absent, alpha and PP cells were restored, and epsilon cells, which were overabundant in the *Nkx2.2^null^*, were significantly reduced (Figure 1A–1L; Figure S1). The partial rescue of the epsilon cells is likely due to the inability of *Neurod1* deletion to restore the balance between the epsilon and beta cell populations, similar to the *Nkx2.2^null^;Neurod1^null^* mice (Figure 1N; Figure S1; [25]). Hormone expression was quantified using real time PCR and cell numbers were determined with morphometric analysis; these analyses confirmed that the observed gene expression and cellular changes were equivalent between the *Nkx2.2^null^;Neurod1^Δpanc^* and the *Nkx2.2^null^;Neurod1^null^* (Figure 1M–1O; Figure S1). Moreover, we confirmed that *Neurod1* was appropriately deleted in mutants and controls (Figure 1P). These data demonstrate that in an *Nkx2.2* null background the deletion of *Neurod1* in the pancreas progenitors phenocopies the *Nkx2.2^null^;Neurod1^null^*.

### In mice lacking Nkx2.2, removal of *Neurod1* from Neurog3+ endocrine progenitor cells restores relative ratios of PP and epsilon cells

**Figure 2:**
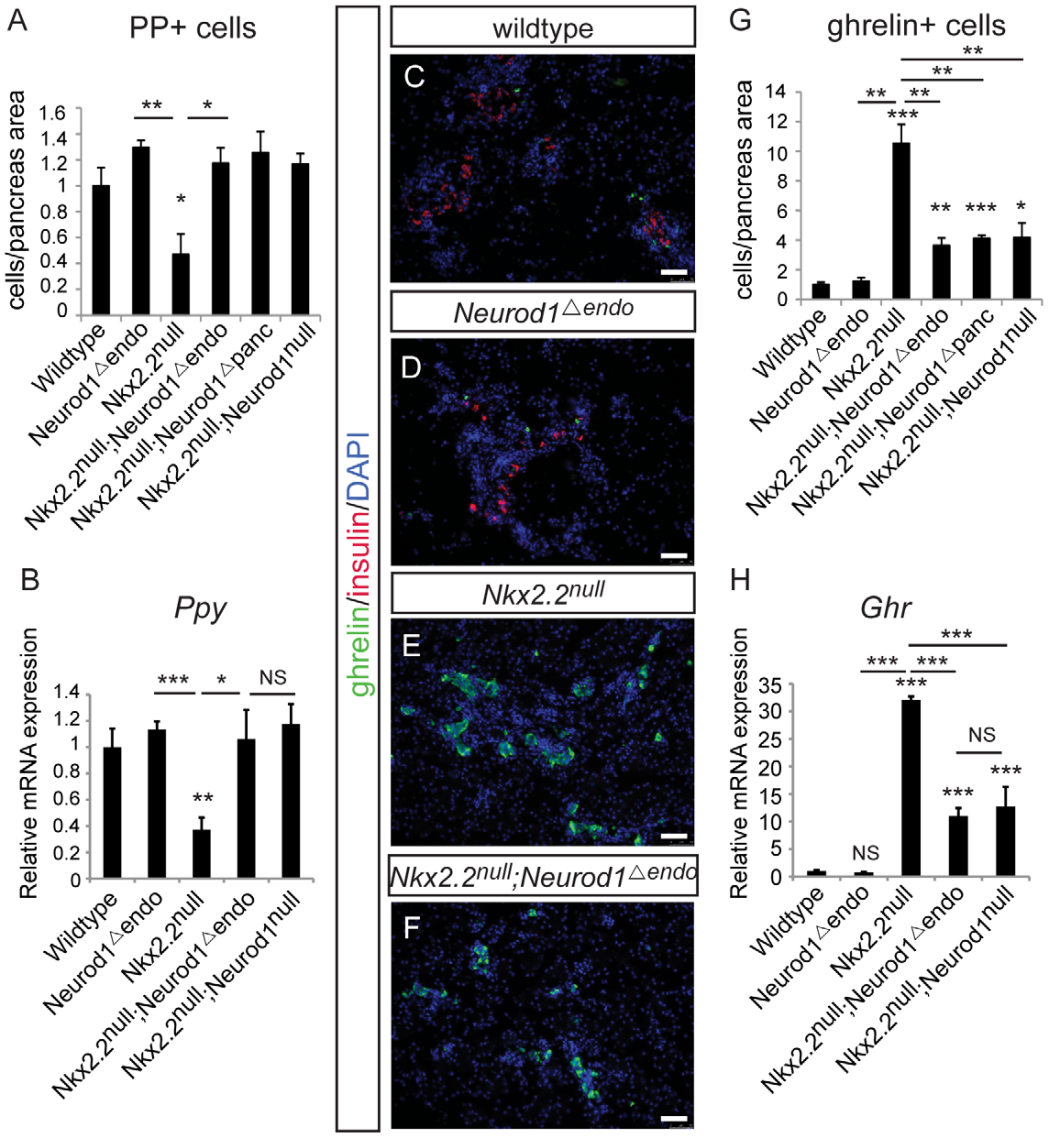
The genetic interaction of Nkx2.2 and Neurod1 is required in the Neurog3+ endocrine cells to specify PP and epsilon cells. Pancreatic polypeptide-expressing PP cells (A) and ghrelin-expressing epsilon cells (G) were quantified by morphometric analysis, comparing wildtype, *Neurod1^Δendo^*, *Nkx2.2^null^*, *Nkx2.2^null^;Neurod1^Δendo^*, *Nkx2.2^null^;Neurod1^Δpanc^*, and *Nkx2.2^null^;Neurod1^null^* at P0. Cell numbers were quantified relative to total pancreas area and displayed normalized to wildtype. Representative sections stained for ghrelin and insulin illustrate the change in ghrelin-expressing cells between genotypes, and the absence of insulin-expressing cells the *Nkx2.2^null^* and *Nkx2.2^null^;Neurod1^Δendo^* (C–F; white bar indicates 50 microns; DAPI marks all nuclei). The expression of *pancreatic polypeptide* (*Ppy*) (B) and *ghrelin* (*Ghr*) (H) was determined for all genotypes by real time PCR (P0; N = 3–7). Relative mRNA expression was normalized to the housekeeping gene, *cyclophilinB*. Data are represented as mean+/−SEM. * p<0.05; ** p<0.01; *** p<0.001.

Given that all hormone-producing endocrine cells are Neurog3-derived [4], [5], [6], we hypothesized that the genetic interaction between Nkx2.2 and Neurod1 would be required within the Neurog3+ endocrine progenitors to allow for the specification of particular hormone+ cell types. Using the *Neurog3-cre* allele [31], we generated an endocrine progenitor cell-specific deletion of *Neurod1* in the *Nkx2.2* null background (*Nkx2.2^−/−^;Neurod1^flox/flox^;Neurog3-cre*, denoted as *Nkx2.2^null^;Neurod1^Δendo^*), and assessed the pancreatic endocrine cell phenotype. To achieve optimal recombination in the Neurog3-expressing precursor population, we used the BAC-derived *Neurog3-cre* allele; Cre is highly co-expressed with Neurog3 in the embryonic pancreas and Cre activity is sufficient to lineage-label all pancreatic endocrine cells in the islet [31]. Importantly, despite the short half-life of Neurog3 protein, we can detect Cre activity in approximately 75% of Neurog3-expressing cells (Figure S2B). Similar to the *Nkx2.2^null^;Neurod1^Δpanc^* and *Nkx2.2^null^;Neurod1^null^* mice, we observed rescue of PP cells (Figure 2A, 2B), and a large reduction of ghrelin+ epsilon cells in the *Nkx2.2^null^;Neurod1^Δendo^* compared with the *Nkx2.2^null^* mice (Figure 2C–2H). As seen in the *Nkx2.2^null^;Neurod1^Δpanc^* and *Nkx2.2^null^;Neurod1^null^* mice, there was no rescue of the insulin-producing beta cell population (Figure 2C–2F; Figure S3). Given this similar phenotype between the *Nkx2.2^null^;Neurod1^null^*, *Nkx2.2^null^;Neurod1^Δpanc^* and *Nkx2.2^null^;Neurod1^Δendo^* we conclude that the genetic interaction between Nkx2.2 and Neurod1 is required in the Neurog3+ cells to permit specification of the PP and epsilon cell populations.

### *Neurod1* deletion from Neurog3+ endocrine progenitors, in an *Nkx2.2* null background, is insufficient to restore the alpha cell population

**Figure 3:**
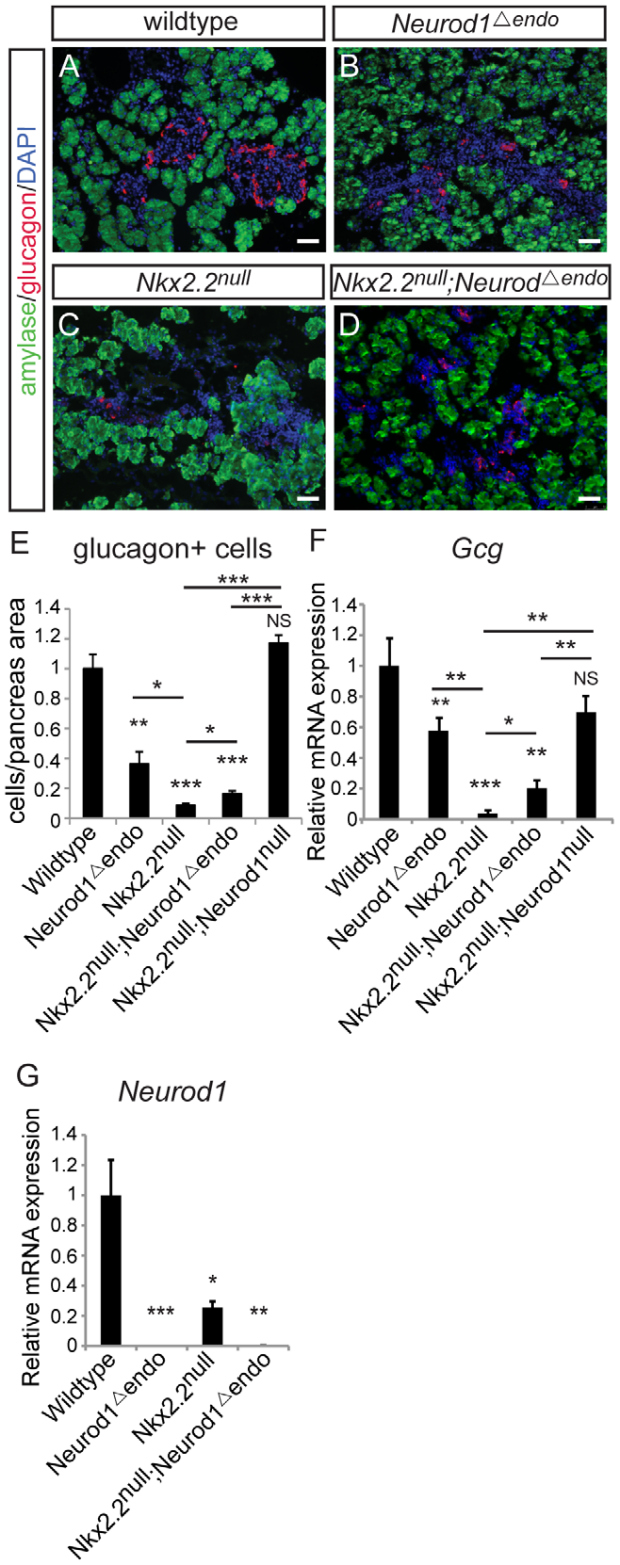
Alpha cells are only minimally restored in the *Nkx2.2^null^;Neurod1^Δendo^*. Pancreatic tissue was analyzed by immunofluorescence for the presence of glucagon-expressing cells at P0, comparing wildtype (A), *Neurod1^Δendo^* (B), *Nkx2.2^null^* (C), and *Nkx2.2^null^;Neurod1^Δendo^* (D). Amylase expression marks exocrine tissue in all genotypes (A–D; white bar indicates 50 microns; DAPI marks all nuclei). Glucagon-expressing alpha cells were quantified by morphometric analysis, relative to total pancreas area and displayed normalized to wildtype (E). The expression of *glucagon* (*Gcg*) (F) and *Neurod1* (G) was measured by real time PCR using RNA from P0 pancreas for all genotypes (N = 3–7). Relative mRNA expression was normalized to the housekeeping gene, *cyclophilinB*. Data are represented as mean+/−SEM. * p<0.05; ** p<0.01; *** p<0.001.

Changes in the beta, PP and epsilon cell populations were identical when *Neurod1* was deleted from either the pancreatic or endocrine progenitors in the absence of Nkx2.2. However, in contrast to the *Nkx2.2^null^;Neurod1^Δpanc^* and the *Nkx2.2^null^;Neurod1^null^*, the glucagon-expressing alpha cell population was only minimally restored in the *Nkx2.2^null^;Neurod1^Δendo^* (Figure 3A–3D). Morphometric analysis (Figure 3E) and real time PCR for *glucagon* expression (Figure 3F) confirmed this observation. We also established that the partial rescue was not due to incomplete deletion of *Neurod1* by *Neurog3-cre*, as *Neurod1* was reduced at an early stage of Neurog3 expression; becoming almost undetectable in the mutant pancreata by P0 (Figure 3G; Figure S4). Taken together, these data suggest that the genetic interaction between Nkx2.2 and Neurod1 in Pdx1+ progenitors, prior to Neurog3+ endocrine progenitor formation, is required for complete alpha cell formation.

### Alpha cells are not recovered with deletion of *Neurod1* from the glucagon+ cells, in the absence of Nkx2.2

**Figure 4:**
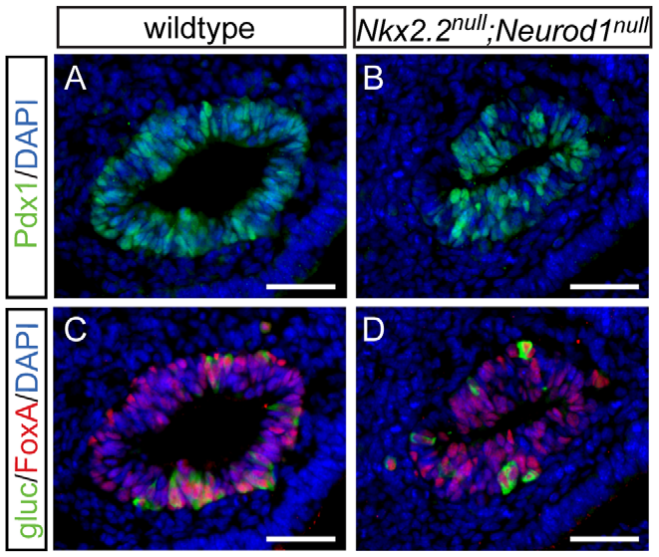
Alpha cells are present in the early pancreatic domain of the *Nkx2.2^null^;Neurod1^null^* double-knockout mouse. Sections from E10.5 wildtype (A) and *Nkx2.2^null^;Neurod1^null^* (B) embryos were stained for Pdx1 to identify the pancreatic domain. Adjacent sections were stained for FoxA and glucagon (gluc), to identify alpha cells in the early pancreatic domain in both the wildtype (C) and *Nkx2.2^null^;Neurod1^null^* (D). White bar indicates 50 microns. DAPI marks all nuclei.

**Figure 5:**
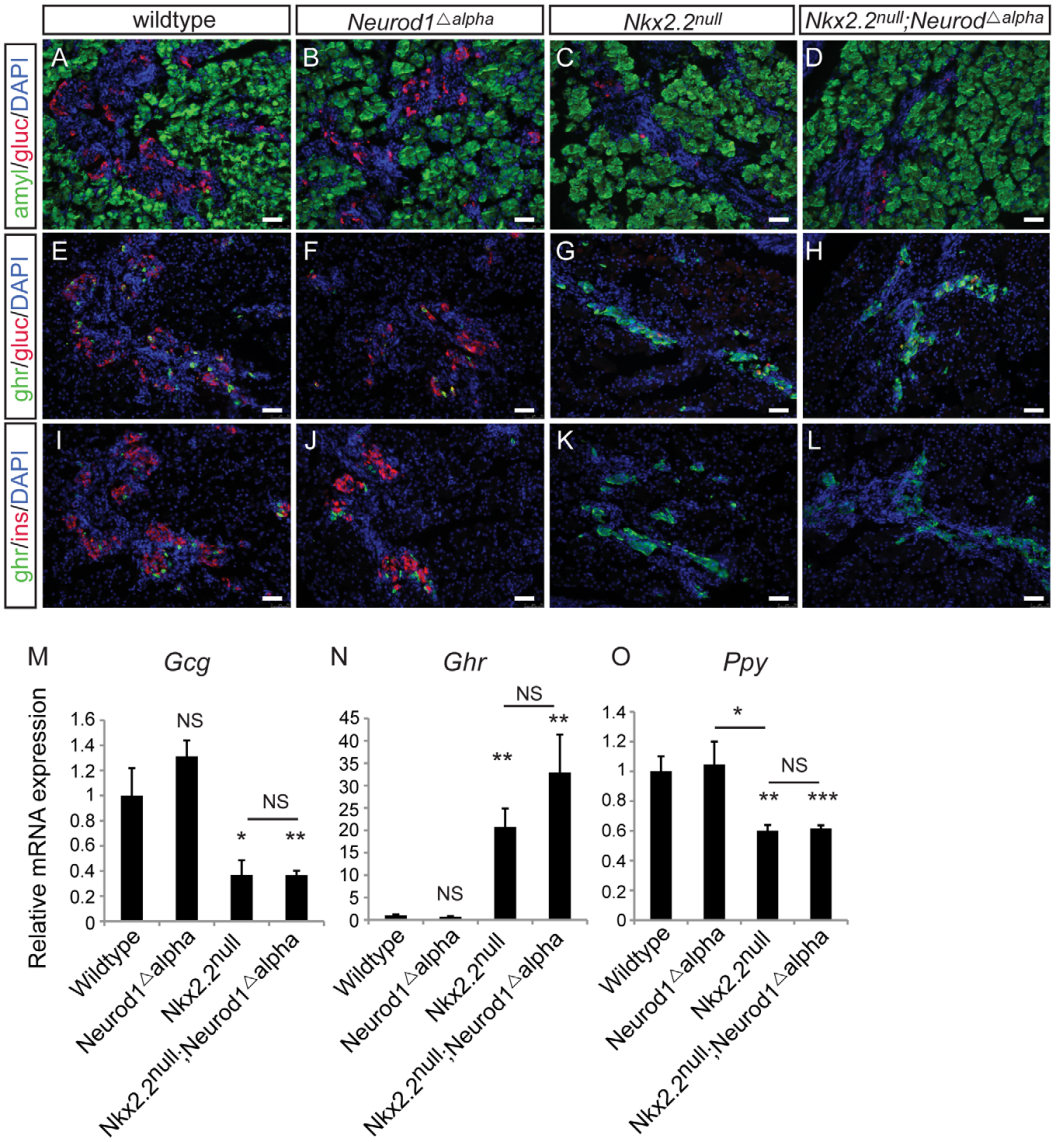
Alpha cells are not rescued with deletion of *Neurod1* in glucagon+ cells, in the absence of Nkx2.2. Pancreatic tissue from wildtype, *Neurod1^Δalpha^*, *Nkx2.2^null^*, and *Nkx2.2^null^;Neurod1^Δalpha^* was analyzed by immunofluorescence for expression of the islet hormones glucagon (gluc), ghrelin (ghr) and insulin (ins) at P0 (A–L; white bar indicates 50 microns; DAPI marks all nuclei). Amylase (amyl) expression marks exocrine tissue in all genotypes (A–D). The quantitative expression of *glucagon* (*Gcg*) (M), *ghrelin* (*Ghr*) (N), and *pancreatic polypeptide* (*Ppy*) (O) was determined by real time PCR using RNA extracted from pancreas (P0; N = 3–7). Relative mRNA expression was normalized to the housekeeping gene, *cyclophilinB*. Data are represented as mean+/−SEM. * p<0.05; ** p<0.01; *** p<0.001.

Data from the *Nkx2.2^null^;Neurod1^Δpanc^* and *Nkx2.2^null^;Neurod1^Δendo^* clearly demonstrate that *Neurod1* must be deleted from the Pdx1+ progenitor population and not the Neurog3+ endocrine progenitor population to allow for complete rescue of alpha cell formation. Furthermore, the simultaneous loss of *Nkx2.2* and *Neurod1* was able to rescue even the earliest glucagon-expressing cell population; the number of glucagon-expressing cells was equivalent between the *Nkx2.2^null^;Neurod1^null^* and wildtype littermate controls at E10.5 (Figure 4A–4D; data not shown), Interestingly, the early glucagon-expressing cells are known to express low levels of Pdx1 (Figure S5; [24]). To determine whether the alpha cell restoration was due to deletion of *Neurod1* specifically from this glucagon+ (Pdx1^low^) population in the absence of Nkx2.2, we deleted *Neurod1* in the glucagon-expressing cells using *Glu-cre* [32] (Figure S2C, S2D). In the *Nkx2.2^−/−^;Neurod1^flox/flox^;Glu-cre* (denoted as *Nkx2.2^null^;Neurod1^Δalpha^*), the complement of all hormone-expressing cells in the pancreas was phenotypically identical to the *Nkx2.2^null^*, as determined by immunofluorescent analysis of islet cell markers (Figure 5A–5L; data not shown) and real time PCR for quantitative hormone expression (Figure 5M–5O; Figure S6). These results suggest that restoration of alpha cells requires the deletion of *Neurod1* in Pdx1+ progenitors that have not yet committed to the glucagon-expressing lineage. We hypothesize that Nkx2.2 represses *Neurod1* in the Pdx1+ cells to give rise to Neurog3+ endocrine progenitor cells that are primed to differentiate into the alpha cell fate.

### Neurod1 is expressed in a subset of Neurog3+ cells and glucagon+ cells

**Figure 6:**
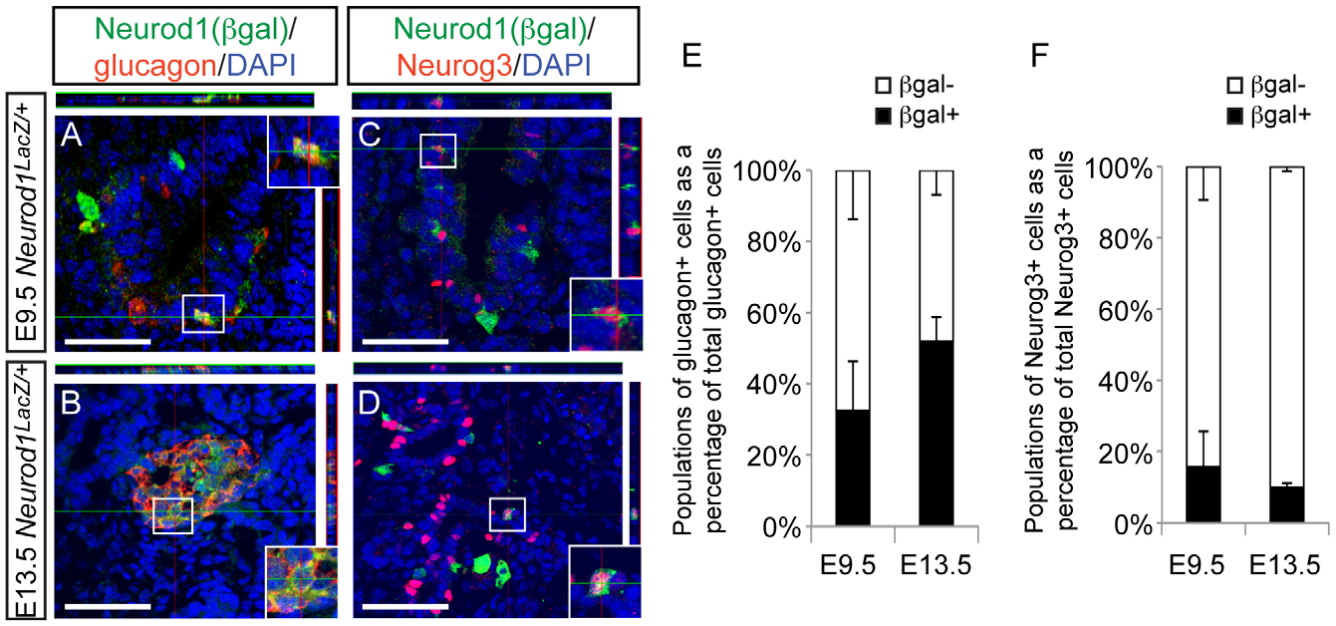
Neurod1 is expressed in a subset of endocrine progenitor cells. Utilizing the *Neurod1:LacZ* knock-in allele (*Neurod1^LacZ/+^*) and immunofluorescence on tissues sections from E9.5 and E13.5 embryos, the expression pattern of Neurod1 (marked by beta-galactosidase; beta-gal) and glucagon (A, B), and Neurod1 and Neurog3 (C, D) was determined. DAPI marks all nuclei. All images are confocal. White bar indicates 50 microns. Boxes denote area magnified for inset, which are +1.75zoom of lower power image. Top and right rectangular panels represent a *Z* projection of at least 10 stack pictures at the level of intersection of the red/green crosshairs. (E) The percentage of each of the populations of glucagon+ cells, or (F) Neurog3+ cells was quantitated at E9.5 and E13.5. Data are represented as mean+/−SEM.

Since *Neurod1* is a downstream target of Neurog3 [33], [34] and the Neurod1 single knockout phenotype does not manifest until the end of gestation [26], it was surprising that manipulation of *Neurod1* within the Neurog3+ endocrine progenitors was not sufficient to rescue the alpha cell fate in the *Nkx2.2* null background. To begin to reconcile these unexpected results, we re-examined when and where Neurod1 was expressed during pancreatic development. It was previously reported that *Neurod1* is expressed at E9.5 in the earliest islet precursors, and is often co-expressed with glucagon [26]. Using the *Neurod1* null mouse, which has a LacZ insertion into the *Neurod1* locus [35], we confirmed the presence of Pdx1+/Neurod1(beta-gal+) cells and glucagon+/Neurod1(beta-gal+) cells in the earliest pancreatic domain (Figure 6A; Figure S7A); however, not all glucagon+ cells were Neurod1+ (Figure 6A, 6E). Consistent with previous reports [28], this pattern was also evident at E13.5 (Figure 6B, 6E) during the stage of pancreas development marked by a major wave of endocrine cell differentiation referred to as the “secondary transition” [36].

*Neurod1* is expressed throughout the epithelial cord region, overlapping extensively with the Neurog3+ precursor cells (Figure S7B, S7C). We used expression of the *Neurod1:LacZ* allele to identify Neurod1 (beta-gal+) cells that co-expressed Neurog3 at E9.5 (Figure 6C) and at E13.5 (Figure 6D). Interestingly, the overlap of Neurog3 and Neurod1 was not exclusive at either age, and a subset of Neurog3+ cells did not express Neurod1 (Figure 6F). We also detected Neurod1 (beta-gal+) expression in a small population of Sox9^low^ cells (Figure S7D–S7F), indicating that Neurod1 expression can be found in cells that are transitioning into Neurog3 precursor cells [37]. Taken together these expression analyses identified heterogeneous populations of Neurog3+ cells and glucagon+ cells based on their expression of Neurod1, and may suggest that the presence or absence of Neurod1 could influence downstream cell fate decisions.

### Nkx2.2 represses *Neurod1* in alpha cells

**Figure 7:**
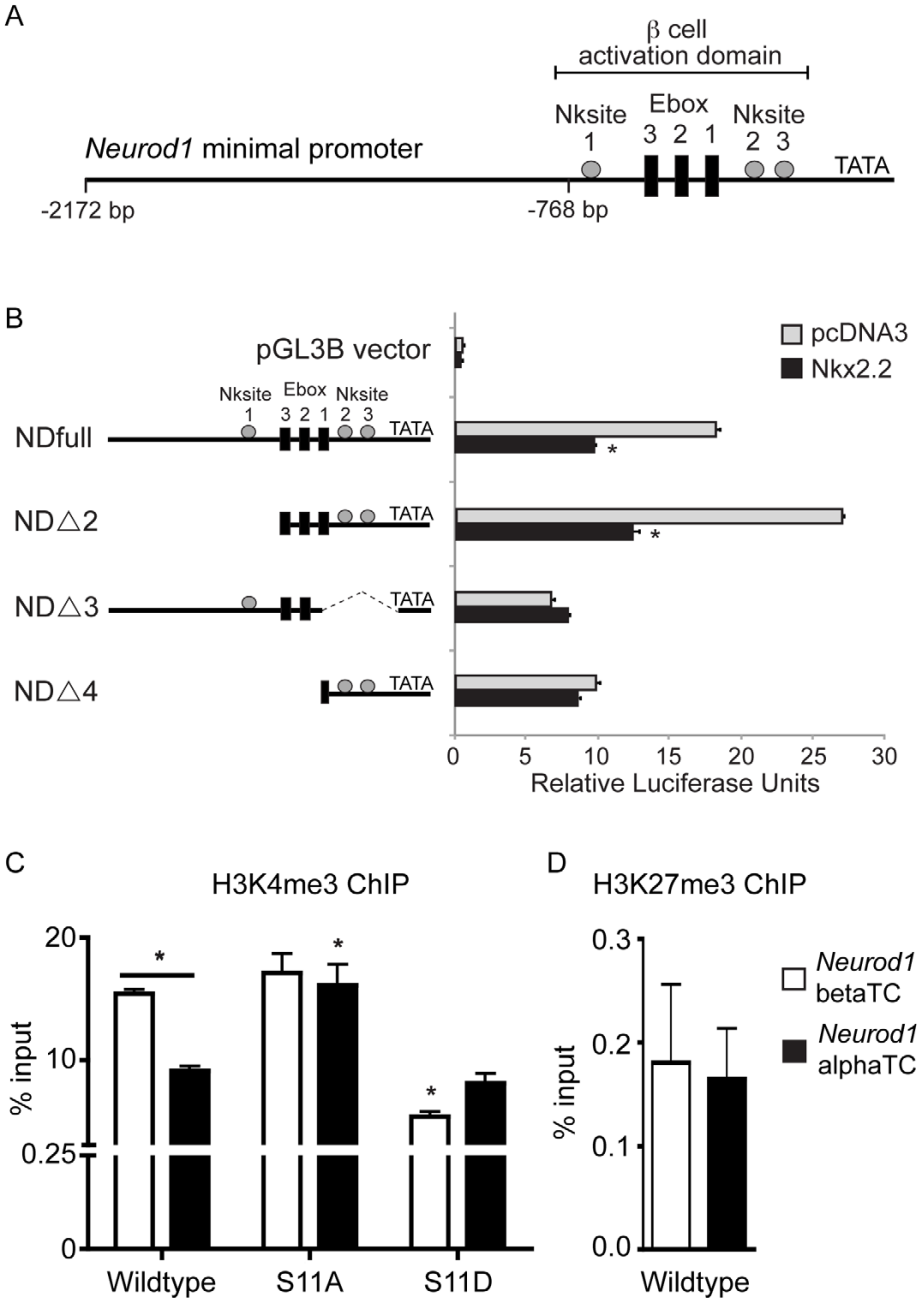
Nkx2.2 represses the *Neurod1* promoter in alphaTC1 cells. (A) Schematic representation of the *Neurod1* minimal promoter, with the areas previously identified to be activated by Nkx2.2 denoted with grey boxes. (B) Luciferase activity was assessed in alphaTC1 cells transfected with *Neurod1* promoter constructs (NDfull, NDΔ2, NDΔ3, NDΔ4) in addition to pcDNA3 alone or Nkx2.2. Nkx2.2-dependent activity was determined based on promoter region deletion. Luciferase activity was determined 48 hours post-transfection. Luciferase readings were normalized to *Renilla* luciferase values. (C) H3K4me3 is enriched in alpha and beta cells, although at significantly lower levels in alpha cells. The Nkx2.2 dephosphorylated mutant (S-11-A) results in a significant increase in H3K4me3 enrichment in alpha cells, comparable to levels observed in beta cell. Conversely, the Nkx2.2 phosphorylation mutant (S-11-D) results in a significant decrease in H3K4me3 in beta cells, comparable to levels in alpha cells. (D) The repressive H3K27me3 mark is not present on the *Neurod1* promoter in alpha or beta cells (*n* = 3). Data was normalized to *Gapdh.* All data are represented as mean+/−SEM. * p<0.05.

Our cumulative data suggest that Nkx2.2 may function to repress *Neurod1* in a subset of Pdx1+ pancreatic progenitor cells to promote specification of the alpha cell fate. We had previously determined that Nkx2.2 directly activates the *Neurod1* promoter in beta cells, which is consistent with the beta cell phenotypes of the single and double knockout mice [12], [26], [28] (Figure 7A). To determine whether Nkx2.2 could also repress *Neurod1* expression in other (non-beta) cell contexts, we analyzed the effect of Nkx2.2 on *Neurod1* expression in alpha cells *in vitro*. Utilizing previously described *Neurod1* promoter deletion constructs [28] we determined that Nkx2.2 repressed the *Neurod1* promoter in alphaTC1 cells, which express Nkx2.2 [28] (Figure 7). Specifically, the repressive activity of Nkx2.2 mapped to the proximal region of the *Neurod1* promoter, which is retained in the NDΔ2 promoter construct (Figure 7B). We also determined that, similar to Nkx2.2-dependent activation of the *Neurod1* promoter in beta cells, Nkx2.2 repression required the presence of at least one of the three Nkx2.2 binding sites; deletion of either region containing these consensus elements (promoter constructs NDΔ3, NDΔ4) resulted in a loss of Nkx2.2 repression (Figure 7B).

To begin to understand how Nkx2.2 mediates differential cell context-specific regulatory activities through the same set of promoter elements, we assessed the ability of Nkx2.2 to recruit specific cofactors and/or modified histones to the *Neurod1* promoter in alpha versus beta cell lines. We previously demonstrated that Nkx2.2 preferentially recruits Grg3 and a large co-repressor complex to the inactive *Arx* promoter in beta cells, but this complex was not present on the same promoter region in alpha cells, where *Arx* was actively transcribed [14]. Surprisingly, neither Grg3 nor HDAC1 were recruited to the *Neurod1* promoter in either alpha or beta cell lines (data not shown), suggesting that Nkx2.2 mediates *Neurod1* regulation through an alternative mechanism. Interestingly however, we determined that histone H3K4me3 preferentially occupied the *Neurod1* promoter in beta cells, and this differential binding was dependent upon the phosphorylation state of Nkx2.2 (Figure 7C). Histone H3K27me3 was not significantly present at the *Neurod1* promoter in either alpha or beta cell lines (Figure 7D). These results suggest that while Nkx2.2 promotes activation of *Neurod1* in beta cells [28], Nkx2.2 appears to prevent the activation of the *Neurod1* promoter in alpha cells. This finding is consistent with the idea that Nkx2.2 is required to prevent expression of *Neurod1* in a subset of Pdx1+ progenitor cells and then maintain this repression in “alpha-cell competent” Neurog3-expressing cells, and subsequently mature alpha cells.

## Discussion

Single deletion mutants have identified the importance of a number of transcription factors for the process of endocrine cell differentiation (reviewed in [38]). Interestingly, very few factors when deleted affect only one islet cell type. Therefore we can deduce that each regulatory protein has multiple roles during development and it is likely that different combinations of these factors must be simultaneously present or absent within the endocrine progenitor cells to permit the specification of alpha, beta, delta, epsilon or PP cells. The generation of compound deletion mutants would assist in deciphering this combinatorial transcription factor code. One such example is the regulatory interaction between Nkx2.2 and the alpha cell transcription factor Arx; simultaneous deletion revealed that these factors differentially cooperate to affect the specification of several islet cell lineages [23], [24]. In this current study, we explore the relative roles of Nkx2.2 and the beta cell transcription factor Neurod1. The single deletion mutants for *Nkx2.2* or *Neurod1* display alterations in several islet cell types [12], [26]; however, these mutants are noted for their severe beta cell phenotypes. In particular, Nkx2.2 and Neurod1 are necessary for beta cell specification and maintenance, respectively [12], [26]. Interestingly, simultaneous deletion of *Nkx2.2* and *Neurod1* did not affect the respective beta cell phenotypes of the single mutants, but rather identified complex genetic interactions between these factors for the specification of alpha, PP and epsilon cells [25]. In this set of experiments, we have determined the cellular locations of the genetic interactions between Nkx2.2 and Neurod1, and have uncovered a possible mechanism for how these transcription factors contribute to the process of alpha cell specification. Given the increasing number of studies identifying transdifferentiation between alpha cells and beta cells [10], [14], [39], refining our understanding of alpha cell development may provide insight into the unique relationship between alpha and beta cells, and ultimately aid in understanding how beta cells develop in both the normal and diseased state.

Knowing that all endocrine cell types are derived from Neurog3-expressing cells [5], [6], we hypothesized that the genetic interaction between Nkx2.2 and Neurod1 would be required in the Neurog3+ endocrine progenitors to specify islet cell fates. In support of this hypothesis, deletion of *Neurod1* from the Neurog3+ endocrine progenitor cells in an *Nkx2.2* null background (*Nkx2.2^null^;Neurod1^Δendo^*) was sufficient to rescue the relative ratios of the ghrelin-expressing epsilon cells and pancreatic polypeptide-expressing PP cells when compared to the *Nkx2.2* null phenotype. This demonstrates that the genetic interaction between Nkx2.2 and Neurod1 is required within the Neurog3+ endocrine progenitor population to permit appropriate specification of the PP and epsilon cell populations. In contrast, although alpha cells were completely rescued in the *Nkx2.2^null^;Neurod1^Δpanc^*, we observed only a minimal restoration of glucagon+ cells in the *Nkx2.2^null^;Neurod1^Δendo^*, suggesting that alpha cell recovery requires the genetic interaction between Nkx2.2 and Neurod1 to occur within the Pdx1+ pancreatic progenitors, prior to Neurog3+ endocrine progenitor cell formation. This finding would support the concept proposed by Degraz and Herrera [7] that the Neurog3+ endocrine progenitors represent a heterogeneous population of unipotential cells that are already committed to become a single hormone-producing cell fate.

If all Neurog3+ progenitors are indeed unipotent, then how do we explain rescue of the PP and ghrelin cell ratios that resulted from manipulating gene expression after the Neurog3+ cells are formed? It is possible that there are both unipotential and multipotential endocrine progenitor populations. Alternatively the “pro-PP” or “pro-ghrelin” Neurog3+ populations may retain more plasticity throughout development. The latter explanation is consistent with the findings of Johansson et al., [9], which demonstrated that as development proceeds the progenitor cells are less competent to produce alpha cells and instead favor the generation of other endocrine cell types. This would suggest that although the alpha cell fate decision can be made at multiple points during development, the ability to generate alpha cells is most robust in the earliest pancreatic progenitors and becomes restricted over time. Alternatively, it is possible that later born progenitors retain a certain degree of plasticity that accounts for their ability to respond to lineage manipulations after Neurog3+ cell specification has occurred.

The inability to rescue alpha cells by simultaneously removing *Nkx2.2* and *Neurod1* from the Neurog3+ precursor population, suggests that the genetic interaction between Nkx2.2 and Neurod1 is required in the Pdx1+ progenitor population, prior to acquisition of Neurog3 expression. However, it remains possible that there is a spectrum of *Neurog3-cre* activity within a Neurog3+ precursor cell, with Cre-based inactivation reaching its peak in the middle or late in the lifespan an individual cell. If this were the case, and the genetic interaction between Nkx2.2 and Neurod1 is required only early in the lifespan of a Neurog3+ precursor to rescue alpha cells, then *Neurog3-cre* activity may occur too late within this population to affect its differentiation potential. Although we are unable to resolve the kinetics of Cre activity in the lifespan of a single cell, we can demonstrate co-expression of Neurog3, Cre and R26R reporter activity, suggesting that although Neurog3 protein expression is transient, Cre is present and active in most of the Neurog3+ population during the time window when Neurog3 is expressed (Figure S2B). Furthermore, published lineage studies using this *Neurog3-cre* allele demonstrated that all endocrine cells of the islet, including the glucagon-expressing alpha cells, are labeled by a Cre-dependent R26R:LacZ reporter [31]. This would suggest that even if alpha cells can only be differentiated from “young” Neurog3+ precursors, there is sufficient Cre activity at this earliest stage during the lifespan of a Neurog3+ cell to genetically label the alpha cell population.

Our failure to recover alpha cells by deleting *Neurod1* in a glucagon-expressing population may also be due to the inefficiency of the *Glu-cre* allele, especially in *Nkx2.2^null^* embryos that have a severe reduction in alpha cell numbers. However, we detected similar levels of *Glu-cre* activity in wildtype and *Nkx2.2^null^* pancreata, which should have been sufficient to permit any possible alpha cell rescue (Figure S2C–S2D; see Materials and Methods). Although caveats exist with the use of Cre/lox technologies, these are currently the best tools available to assess spatial and temporal protein function.

Interestingly, we do observe some rescue of alpha cells in the *Nkx2.2^null^; Neurod1^Δendo^* embryos. This could be due to deletion of *Neurod1* in a subset of Neurog3+ progenitors that have not yet become restricted in their ability to differentiate into alpha cells. Alternatively, the glucagon-expressing cells recovered in the *Nkx2.2^null^;Neurod1^Δendo^* may represent alpha cells that form independent of Neurog3 function; such an alpha cell population has been previously documented [40], [41]. On the other hand, the recovered alpha cells may actually represent a distinct subpopulation of glucagon-expressing cells that express Neurod1, which would be consistent with our identification of a subpopulation of glucagon+/Neurod1+ cells. While these explanations are not mutually exclusive, the identification of unique alpha cell markers and the generation of genetic tools utilizing these markers, would be necessary to clarify the existence of subpopulations of alpha cells, as well as the factors involved in the generation of these distinct populations.

**Figure 8:**
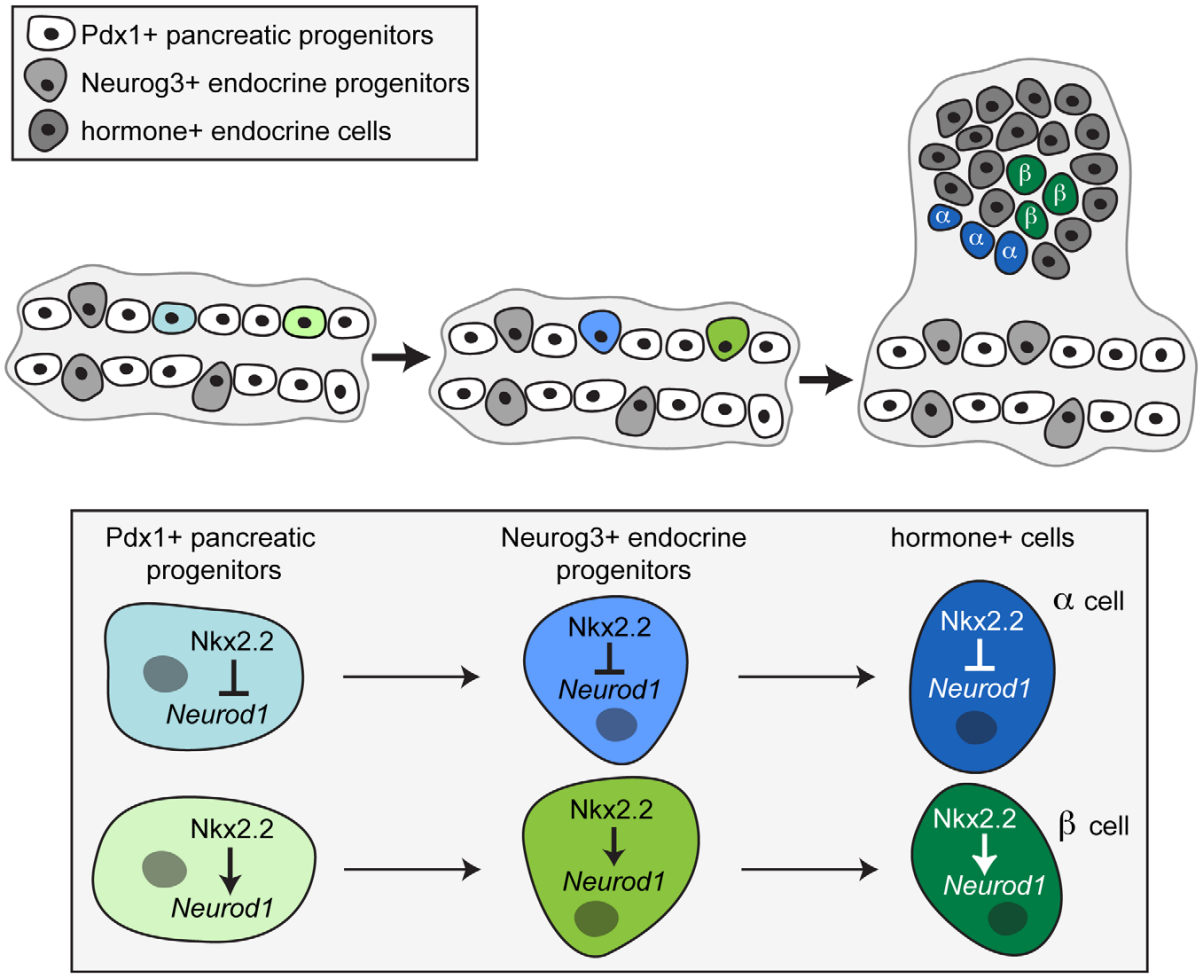
A proposed model for the involvement of Nkx2.2 and Neurod1 in alpha and beta cell specification. Taking into account both our *in vivo* and *in vitro* data, we propose that specific combinations of transcription factors acting in the progenitor cells within the early pancreatic epithelium set up the competency of the unipotent endocrine progenitors to become specific islet cell types. Specifically, we propose a model whereby Nkx2.2 must repress *Neurod1* in a Pdx1+ progenitor, and this repression maintained in the Neurog3+ endocrine progenitor, thereby permitting glucagon-expressing alpha cell specification. Conversely, activation of *Neurod1* by Nkx2.2 permits beta cell formation.

Our findings also suggest that Nkx2.2 must regulate *Neurod1* differentially in the Pdx1+ progenitor population in the early pancreatic epithelium in order to initiate the specification of different populations of Neurog3-expressing cells. In particular, the prevention of *Neurod1* activation by Nkx2.2 would result in alpha cell formation, while the activation of *Neurod1* by Nkx2.2 results in beta cell formation (Figure 8). This is compatible with our discovery that not all Neurog3+ cells express Neurod1, and further supports the idea that the Neurog3+/Nkx2.2+/Neurod1+ cells most likely become beta cells, whereas Neurog3+/Nkx2.2+/Neurod1− cells would become alpha cells. Ideally, we would test this hypothesis by quantifying the increase in the number of Pdx1+/Neurod1+ pancreas progenitors and/or Neurog3+/Neurod1+ endocrine progenitors expected to be observed in the *Nkx2.2^null^* pancreas; however, this analysis is confounded by the simultaneous loss of the Neurod1+ pro-beta cell progenitor populations in the *Nkx2.2^null^* pancreas. Instead, we used an *in vitro* approach to determine whether it was possible for Nkx2.2 to differentially regulate the *Neurod1* promoter in different cellular contexts. We had previously demonstrated that *Neurod1* is activated by the cooperative binding of Nkx2.2 and Neurog3 specifically in beta cells [28]. Given the lack of availability of an appropriate pancreatic progenitor cell line, we reasoned that a genetic interaction between Nkx2.2 and Neurod1 that was initiated in a “pro-alpha cell” progenitor would be maintained in the mature alpha cell. We utilized alphaTC1 cells, which express Nkx2.2 [28], to demonstrate that Nkx2.2 prevents activation of *Neurod1* in alpha cells. Highlighting the complexity of gene regulation, the cell type specific regulation of *Neurod1* by Nkx2.2 appears to function through a mechanism that is different from Nkx2.2 regulation of the *Arx* gene [14]. This may reflect the mechanism by which Nkx2.2 functions as an activator and a repressor in the same cell type and/or the presence or absence of cell-specific co-regulatory proteins. As we gain the molecular tools to study transcriptional and epigenetic mechanisms in purified primary pancreatic cell populations, we hope to elucidate the complex regulatory interactions that are required to form and maintain appropriate islet-cell specific gene expression.

While the process of endocrine specification likely requires the concerted action of many factors, our data suggest a mechanism that involves the differential regulation of *Neurod1* by Nkx2.2 in the Pdx1+ pancreatic progenitor cells to direct the subsequent endocrine progenitors to become specific islet cell types. The generation of tools to identify, separate and analyze different subpopulations of Neurog3+ progenitor cells would conclusively determine whether each hormone+ endocrine cell type is derived from a specific unipotent subpopulation of endocrine progenitor cells, each bearing a unique gene profile.

Using the pancreas as a model system, our study has provided a prime example of how lineage decisions are established in the developing epithelium. The cooperative action of multiple transcription factors within the early progenitor cells can dictate the fate of subsequent cell lineages. Altering the regulation or complement of this set of factors within the progenitor populations can ultimately skew cell lineage specification. These data have important implications for the current efforts to generate pancreatic cells *in vitro* for therapeutic use in diabetic patients. Understanding the cooperative transcription factor code will make it possible to initiate the appropriate program in the Pdx1+ pancreatic progenitor cells necessary to correctly prime the Neurog3+ endocrine progenitor cells and generate pools of functional, single hormone-expressing islet cell types *in vitro*. 

## Materials and Methods

### Mice

All experiments involving mice were approved by the Columbia University Institutional Animal Care and Use Committee and performed in accordance with the National Institutes of Health guidelines for the care and use of animals. All mouse strains were previously generated, and were bred and maintained on an outbred Black Swiss background (NTac:NIHBS, Taconic). Cell-specific *Neurod1* null mice were generated by intercrossing *Neurod1^tm1Kan^* (*Neurod1^flox/flox^*; [42]) and either *Tg(Ipf1-cre)1Tuv* (*Pdx1-cre*; [29]), *Tg(Neurog3-cre)C1Able* (*Neurog3-cre*; [31]), or *Glu-cre* ([32]) mice. *Neurod1^flox/flox^;Pdx1-cre* and *Neurod1^flox/flox^;Neurog3-cre* mice died postnatal, similar to the *Neurod1* null (data not shown; [30]). Certain experiments required the use of either *Gt(ROSA)26Sor^tm9(CAG-tdTomato)Hze^* (*R26R:Tomato*; [43]) or *Gt(ROSA)26Sor^tm1Sor^* (*R26R:LacZ*; [44]) reporter alleles. The *Pdx1-cre* will delete *Neurod1* in all pancreatic progenitor cells; however, the Pdx1 expression domain also includes a portion of the stomach and the duodenum [45], [46]. We and others have previously reported the early and relatively non-mosaic activity of the *Pdx1-cre* allele ([29], [47]; Figure S2A). Previous characterization of the *Neurog3-cre* allele demonstrated almost complete co-expression of Neurog3 and Cre and sufficient Cre activity to lineage label all endocrine cells within an islet [31]. Consistent with this published analysis, quantification of cells co-expressing Neurog3 and the LacZ reporter in a *Neurog3-Cre;R26R:LacZ* E15.5 embryo indicated 74.82% Cre efficiency (268 Neurog3+ beta− gal+/349 total Neurog3+ cells; calculations were performed as described below (Figure S2B). Similar assessment of the *Glu-cre* mice demonstrated that the *Glu-cre* allele is active in approximately 30–35% of alpha cells; notably this degree of activity is unchanged in the *Nkx2.2^null^* background, despite the overall reduction in alpha cell numbers (Figure S2C, S2D).

The heterozygous mice (*Neurod1^flox/+^;Pdx1-cre*) were crossed to *Nkx2-2^tm1Jlr^* knock-in mice [12] to generate compound heterozygotes. Embryos were collected from timed matings between *Nkx2.2^+/−^;Neurod1^flox/+^;Pdx1-cre* and *Nkx2.2^+/−^;Neurod1^flox/^* ^flox^ or Nkx2.*2^+/−^;Neurod1^flox/+^;Neurog3-cre* and *Nkx2.2^+/−^;Neurod1^flox/^* ^flox^ or *Nkx2.2^+/−^;Neurod1^flox/+^;Glu-cre* and *Nkx2.2^+/−^;Neurod1^flox/^* ^flox^ mice. Noon on the day of appearance of a vaginal plug was considered embryonic day (E) 0.5. The experimental genotypes of wildtype, *Nkx2.2^−/−^* (*Nkx2.2^null^*), *Neurod1^flox/flox^;Pdx1-cre* (*Neurod1^Δpanc^*), *Nkx2.2^−/−^;Neurod1^flox/flox^;Pdx1-cre* (*Nkx2.2^null^;Neurod1^Δpanc^*), *Neurod1^flox/flox^;Neurog3-cre* (*Neurod1^Δendo^*), *Nkx2.2^−/−^;Neurod1^flox/flox^;Neurog3-cre* (*Nkx2.2^null^;Neurod1^Δendo^*), *Neurod1^flox/flox^;Glu-cre* (*Neurod1^Δalpha^*), and *Nkx2.2^−/−^;Neurod1^flox/flox^;Glu-cre* (*Nkx2.2^null^;Neurod1^Δalpha^*) were studied. Litters were assessed at postnatal day (P) 0. For expression studies, the *Neurod1^tm1Jle^* LacZ knock-in (*Neurod1^LacZ/+^* or *Neurod1^null^*) [35] was used (also in combination with the *Nkx2.2^null^* thereby producing *Neurod1^null^;Nkx2.2^null^* double knockout embryos; DKO), and embryos were assessed at E9.5, E10.5, E13.5 and P0. All embryo dissections were carried out in cold PBS, using a dissecting microscope (Leica MZ8). A portion of each embryonic tail or yolk sac was detached from the embryo, digested with proteinase K, and DNA extracted for genotyping purposes. Genotyping was carried out with standard conditions and primers as previously described [12], [29], [31], [32], [35], [42]. 

### Real-time PCR

Pancreas was dissected from each embryo and stored in RNAlater (Ambion) until RNA was extracted using the NucleoSpin RNAII Kit (Clontech). Subsequently, cDNA was made with equal amounts of RNA for each sample (Superscript III Kit, Invitrogen, CA). Real time PCR was performed using TaqMan gene expression assays (Applied Biosystems) for *glucagon* (Mm00801712_m1), *ghrelin* (Mm00445450_m1), *somatostatin* (Mm00436671_m1), *insulin1* (Mm01950294_s1), *insulin2* (Mm00731595_gH), *pancreatic polypeptide* (Mm00435889_m1) and *Neurod1* (Mm01280117_m1). *CyclophilinB* was used as a control housekeeping gene, and was assayed using a probe and primer set previously described [25]. A standard two-step real time PCR program was used for all genes assessed, with an annealing temperature of 61°C and 40 cycles of amplification (CFX96 RealTime System C1000 Thermal Cycler, Biorad). All gene expression values were normalized to the internal control gene, *cyclophilinB*, and relative quantification was performed using a standard curve from embryonic age-matched cDNA. Statistical analyses were conducted with Prism Software (GraphPad Software, La Jolla, CA) using both the Mann-Whitney test and the Student t-test. Equivalent results were obtained; t-test results were reported in all Figures.

### Immunofluorescence

Immunofluorescence was performed according to standard protocols, on E9.5, E10.5, E13.5, E15.5 and P0 whole embryos that were embedded in OCT, after fixation with 4% PFA and cryopreservation in 30% sucrose. Transverse frozen sections (8 µm) were cut and mounted on glass slides. Sections were stained with rabbit α-ghrelin (1∶800; Phoenix Pharmaceuticals, CA), goat α-ghrelin (1∶800; Santa Cruz), guinea pig α-glucagon (1∶1000; Linco/Millipore, MA), guinea pig α-insulin (1∶1000; Millipore), rabbit α-insulin (1∶1000; Cell Signaling Technology), rabbit α-somatostatin (1∶200; Phoenix Pharmaceuticals), rabbit α-pancreatic polypeptide (1∶200; Zymed), rabbit α-amylase (1∶1000; Sigma), rabbit α-Pdx1 (1∶1000; Millipore), guinea pig α-Pdx1 (1∶500; BCBC), rabbit α-Neurog3 (1∶500; BCBC), goat α-Neurog3 (1∶500; BCBC), goat α-FoxA (1∶1000; Santa Cruz), rabbit α-sox9 (1∶500; Chemicon), and chicken α-beta-galactosidase (1∶250; Abcam). Donkey α-guinea pig-Cy2, -Cy3 or -Cy5, α-rabbit-Cy2 or -Cy3, α-chicken-Cy3, and α-goat Cy2 or -Cy5 secondary antibodies were used (1∶400, Jackson ImmunoResearch). DAPI (1∶1000; Invitrogen) was applied for 30 minutes following secondary antibody incubation. Images were acquired on a Leica DM5500 or Leica 510 confocal microscope. Morphometric analysis was performed by immunostaining every 10^th^ section throughout each embryo (N = 3 or 4 for each genotype). For quantification of individual hormone-expressing cells at P0, cell number was assessed versus total pancreas as defined by amylase area. For quantification of hormone-expressing cells at E10.5, cell number was assessed versus total pancreas as defined by Pdx1 area. Pancreas area was calculated using ImagePro software.

### RNA *in situ* hybridization

RNA *in situ* hybridization was performed on 8 µm sections mounted on glass slides as previously described [25] using an antisense riboprobe transcribed from linearized plasmid. The riboprobe for *Neurod1* was generated from the plasmid pCS2:MTmNeuroD1 (J. Lee). RNA *in situ* hybridization was performed on pancreas tissue sections from *Neurod1^Δendo^* and wildtype littermate controls at E10.5 and *Neurog3-cre;R26R^LacZ^* at E15.5.

### Luciferase reporter assays

The *Neurod1*-2.2 kb minimal promoter was fused to the firefly luciferase open reading frame in the pGL3 Basic vector (Promega). The alphaTC1 cells were grown in 12-well plates. The design of all *Neurod1* promoter deletion constructs and the transfection conditions were previously described [28]. Firefly luciferase readings were normalized to *Renilla* luciferase values. A Student t-test was performed to determine significance.

### Chromatin immunoprecipitation

Point mutations were made to 3xmyc-tagged Nkx2.2 cDNA using the QuickChange II Site Directed Mutagenesis kit (Agilent Technologies) with the following primers S-11-A: (FWD) CAACACAAAGACGGGGTTTGCTGTCAAGGACATCTTGGAC, (REV) GTCCAAGATGTCCTTGACAGCAAACCCCGTCTTTGTGTTG; S-11-D: (FWD) CAACACAAAGACGGGGTTTGATGTCAAGGACATCTTGGAC, (REV) GTCCAAGATGTCCTTGACATCAAACCCCGTTTTGTGTTG. Wild type or mutated Nkx2.2 cDNA encoding a triple myc epitope tag (250 ng) was transfected into betaTC6 or alphaTC1 cells using X-treme gene HP (Roche) according to manufacturer's protocol. Chromatin was prepared using the ChIP-IT express kit (Active Motif). Immunoprecipitation protocol was modified from Tuteja et al. [48]. In brief, immunoprecipitation was performed using the isolated chromatin diluted in ChIP dilution buffer with 5 micrograms of either mouse anti-H3K27me3 (Abcam) or mouse anti-H3K4me3 (Abcam) antibodies while rotating overnight at 4°C. The following day antibody/chromatin complexes were pulled down using ChIP grade protein G magnetic beads (Cell Signaling). After washing, antibody/chromatin complexes were eluted from the beads and allowed to rotate at room temperature for 15 minutes. NaCl (5 micromolar) was added to the eluate and incubated at 65°C overnight. The following day Tris-HCl (1 M, pH 7.5), EDTA (0.5 M) and proteinase K (10 mg/mL) were added and allowed to incubate at 37°C for 1 hour. Samples were then purified using the QIAquick PCR purification kit (Qiagen). Quantitative analysis of ChIP products was performed using SYBR Green fluorescence with primers for *Gapdh* (FWD – CTCCACGACATACTCAGCACC; REV – TCAACGGCACAGTCAAGGC) or *Neurod1* (FWD – AAAGGGTTAATCTCTCCTGCGGGT; REV - CATGCGCCATATGGTCTTCCCGGT).

## Supporting Information

Figure S1 Morphometric and expression analysis of the *Nkx2.2^null^;Neurod1^Δpanc^*. Glucagon-expressing alpha cells (A), ghrelin-expressing epsilon cells (B) and pancreatic polypeptide-expressing PP cells (C) were quantified by morphometric analysis, comparing wildtype, *Neurod1^Δpanc^*, *Nkx2.2^null^*, *Nkx2.2^null^;Neurod1^Δpanc^*, and *Nkx2.2^null^;Neurod1^null^* at P0. Cell numbers were quantified relative to total pancreas area and displayed normalized to wildtype (N = 3–4). The quantitative expression of *insulin1* (*Ins1*) (D), *insulin2* (*Ins2*) (E), and *somatostatin* (*Sst*) (F) was determined by real time PCR using RNA extracted from wildtype, *Neurod1^Δpanc^*, *Nkx2.2^null^*, *Nkx2.2^null^;Neurod1^Δpanc^*, and *Nkx2.2^null^;Neurod1^null^* pancreas (P0; N = 3–8). Relative mRNA expression was normalized to the housekeeping gene, *cyclophilinB*. Data are represented as mean+/−SEM. * p<0.05; ** p<0.01; *** p<0.001.
(TIF)

Figure S2 Expression analysis in the *Pdx1-cre*, *Neurog3-cre* and *Glu-cre* alleles. A small population of Neurog3-expressing cells at E12.5 was observed to not co-express beta-gal (A). Individual channels were separated in side panel to better visualize the Neurog3 cells that do not express beta-gal. In e15.5 pancreata containing *Neurog3-cre* and the *R26R;LacZ* reporter allele, the majority Neurog3-expressing cells also express beta-gal, a marker of cre activity (B). Cells expressing Neurog3, Cre, and beta-gal were also observed, identifying that both cre expression and cre activity are present within Neurog3-expressing cells (C; inset). Using the *R26R:Tomato* reporter allele, *Glu-cre* activity was assessed in both the wildtype (D) and *Nkx2.2^null^;Neurod1^Δalpha^* (E). The glucagon+ cells were not rescued in this compound mutant, but this was not due to a lack of cre activity from the *Glu-cre* allele. Boxes denote magnified areas (+1.75zoom of low power image). White bars indicate 50 microns.
(TIF)

Figure S3 *Insulin* expression in the *Nkx2.2^null^;Neurod1^Δendo^*. The quantitative expression of *insulin1* (*Ins1*) (A) and *insulin2* (*Ins2*) (B) was determined by real time PCR using RNA extracted from wildtype, *Neurod1^Δendo^*, *Nkx2.2^null^*, and *Nkx2.2^null^;Neurod1^Δendo^* pancreas (P0; N = 3–7). Relative mRNA expression was normalized to the housekeeping gene, *cyclophilinB*. Data are represented as mean+/−SEM. * p<0.05; ** p<0.01; *** p<0.001.
(TIF)

Figure S4 *Neurod1* inactivation by *Neurog3-cre* in the early pancreatic bud. RNA *in situ* hybridization on pancreas sections from E10.5 wildtype (A) and *Neurod1^Δendo^* (C) embryos identified a reduction in *Neurod1* by *Neurog3-cre* even at this early stage of development. Adjacent tissue sections were stained for Pdx1 (B, D) to identify the pancreas area (encircled with dashed lines). White bar indicates 50 microns. DAPI marks all nuclei.
(TIF)

Figure S5 Alpha cells express low levels of Pdx1. A sagittal section through the dorsal pancreas of a wildtype E10.5 embryo was stained for Pdx1 and glucagon. Glucagon+ cells were observed to express low levels of Pdx1. Box denotes area magnified for inset but was imaged without DAPI; +1.75 zoom of low power image). White bar indicates 50 microns. DAPI marks all nuclei.
(TIF)

Figure S6 Insulin expression in the *Nkx2.2^null^;Neurod1^Δalpha^*. The quantitative expression of *insulin1* (*Ins1*) (A) and *insulin2* (*Ins2*) (B) was determined by real time PCR using RNA extracted from wildtype, *Neurod1^Δalpha^*, *Nkx2.2^null^*, and *Nkx2.2^null^;Neurod1^Δalpha^* pancreas (P0; N = 3–7). Relative mRNA expression was normalized to the housekeeping gene, *cyclophilinB*. Data are represented as mean+/−SEM. * p<0.05; ** p<0.01; *** p<0.001.
(TIF)

Figure S7 Neurod1 expression at specific developmental timepoints. Utilizing the *Neurod1:LacZ* knock-in allele (*Neurod1^LacZ/+^*) and immunofluorescence on tissues sections, Neurod1 (marked by beta-galactosidase; beta-gal) cells were identified to co-express Pdx1 at E9.5 (A). The overlap of *Neurod1* and Neurog3 expression was identified at E15.5 by RNA *in situ* hybridization for *Neurod1* (B) and immunofluorescent staining of Neurog3 (C) on the adjacent tissue section from a *Neurog3-cre;R26R:LacZ* embryo. A subset of Neurod1 cells that co-express Sox9 were also identified at E13.5 (D); the Sox9 (E) and beta-gal (F) channels were separated to visualize co-expressing cells more clearly. White bars indicate 50 microns. Boxes denote area magnified for inset, which are +1.75 zoom of lower power image.
(TIF)
