# Supplementary figures and images for: Regulation of Neurod1 Contributes to the Lineage Potential of Neurogenin3+ Endocrine Precursor Cells in the Pancreas
Source: PLoS Genet. 2013 Feb 7;9(2):e1003278. doi: 10.1371/journal.pgen.1003278 (PMC3567185; doi:10.1371/journal.pgen.1003278)

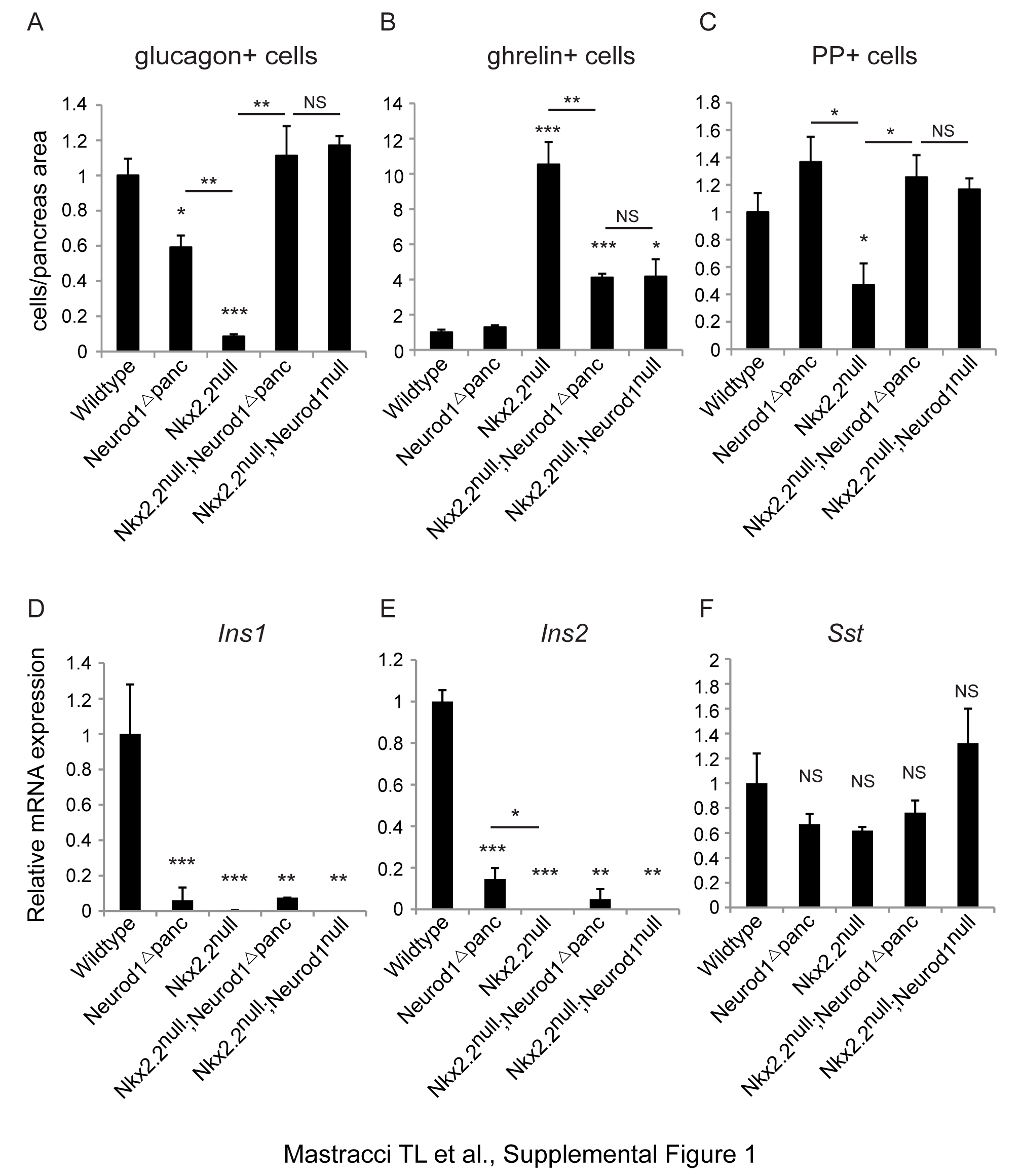

Supplement: Figure S1 — Morphometric and expression analysis of the Nkx2.2null;Neurod1Δpanc. Glucagon-expressing alpha cells (A), ghrelin-expressing epsilon cells (B) and pancreatic polypeptide-expressing PP cells (C) were quantified by morphometric analysis, comparing wildtype, Neurod1Δpanc, Nkx2.2null, Nkx2.2null;Neurod1Δpanc, and Nkx2.2null;Neurod1null at P0. Cell numbers were quantified relative to total pancreas area and displayed normalized to wildtype (N = 3–4). The quantitative expression of insulin1 (Ins1) (D), insulin2 (Ins2) (E), and somatostatin (Sst) (F) was determined by real time PCR using RNA extracted from wildtype, Neurod1Δpanc, Nkx2.2null, Nkx2.2null;Neurod1Δpanc, and Nkx2.2null;Neurod1null pancreas (P0; N = 3–8). Relative mRNA expression was normalized to the housekeeping gene, cyclophilinB. Data are represented as mean+/−SEM. * p<0.05; ** p<0.01; *** p<0.001. (TIF) [file pgen.1003278.s001.tif]

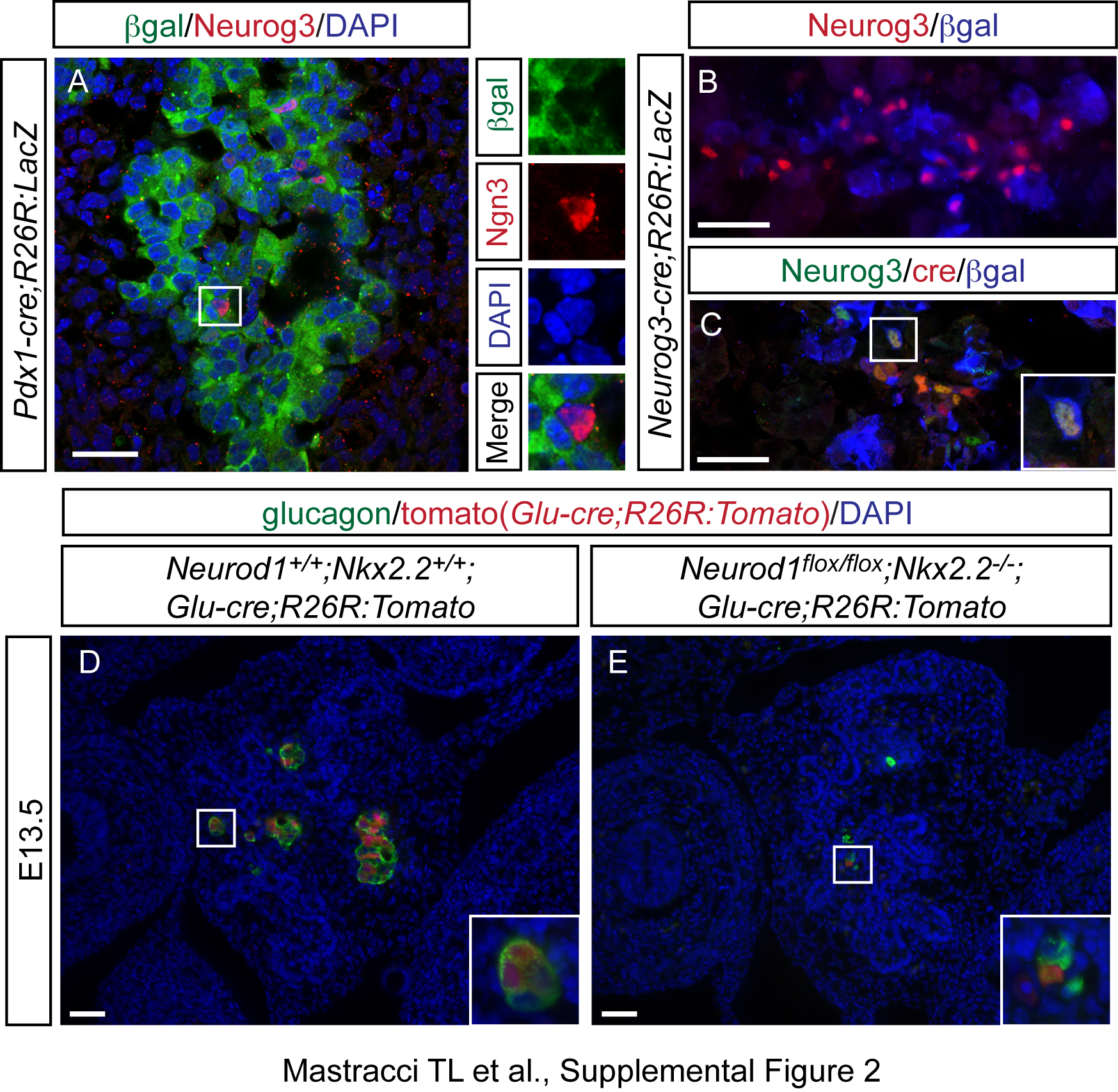

Supplement: Figure S2 — Expression analysis in the Pdx1-cre, Neurog3-cre and Glu-cre alleles. A small population of Neurog3-expressing cells at E12.5 was observed to not co-express beta-gal (A). Individual channels were separated in side panel to better visualize the Neurog3 cells that do not express beta-gal. In e15.5 pancreata containing Neurog3-cre and the R26R;LacZ reporter allele, the majority Neurog3-expressing cells also express beta-gal, a marker of cre activity (B). Cells expressing Neurog3, Cre, and beta-gal were also observed, identifying that both cre expression and cre activity are present within Neurog3-expressing cells (C; inset). Using the R26R:Tomato reporter allele, Glu-cre activity was assessed in both the wildtype (D) and Nkx2.2null;Neurod1Δalpha (E). The glucagon+ cells were not rescued in this compound mutant, but this was not due to a lack of cre activity from the Glu-cre allele. Boxes denote magnified areas (+1.75zoom of low power image). White bars indicate 50 microns. (TIF) [file pgen.1003278.s002.tif]

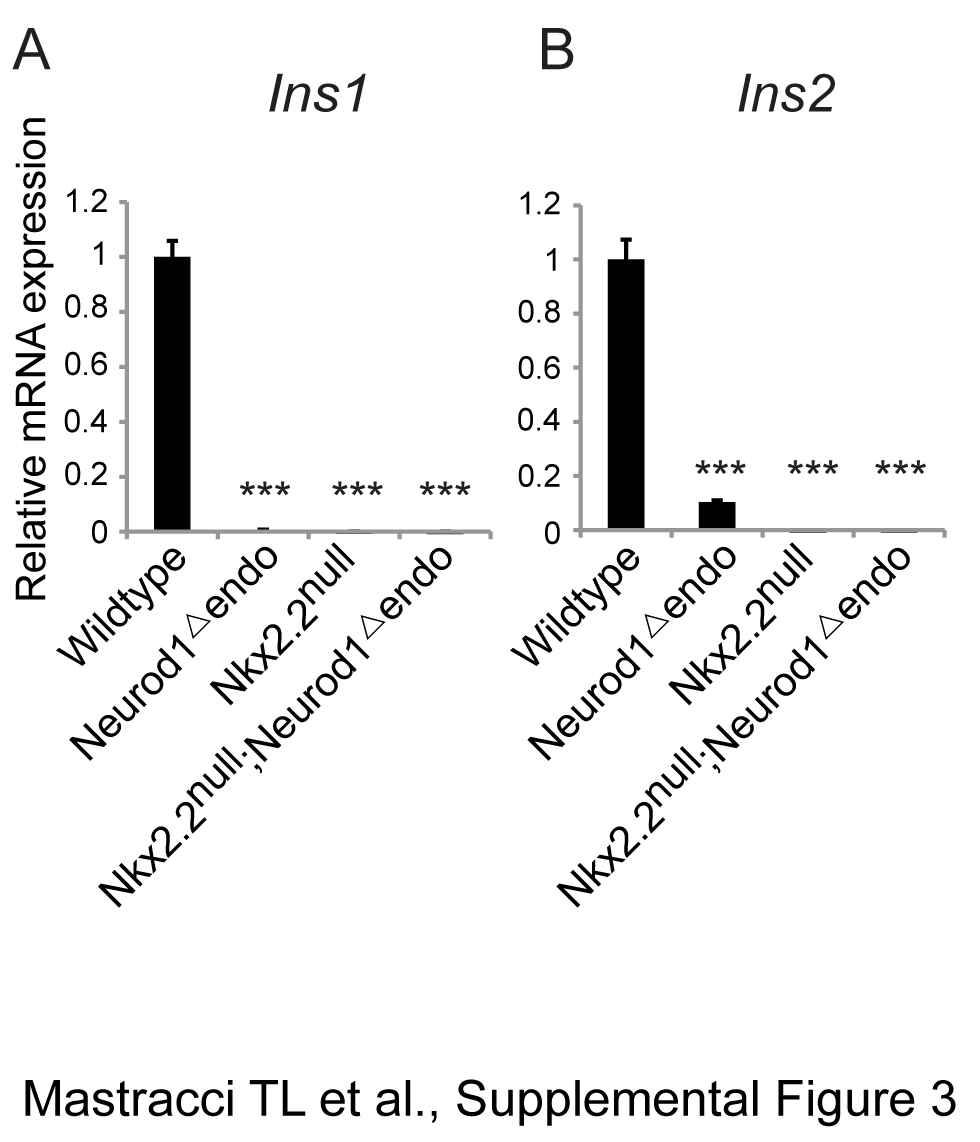

Supplement: Figure S3 — Insulin expression in the Nkx2.2null;Neurod1Δendo. The quantitative expression of insulin1 (Ins1) (A) and insulin2 (Ins2) (B) was determined by real time PCR using RNA extracted from wildtype, Neurod1Δendo, Nkx2.2null, and Nkx2.2null;Neurod1Δendo pancreas (P0; N = 3–7). Relative mRNA expression was normalized to the housekeeping gene, cyclophilinB. Data are represented as mean+/−SEM. * p<0.05; ** p<0.01; *** p<0.001. (TIF) [file pgen.1003278.s003.tif]

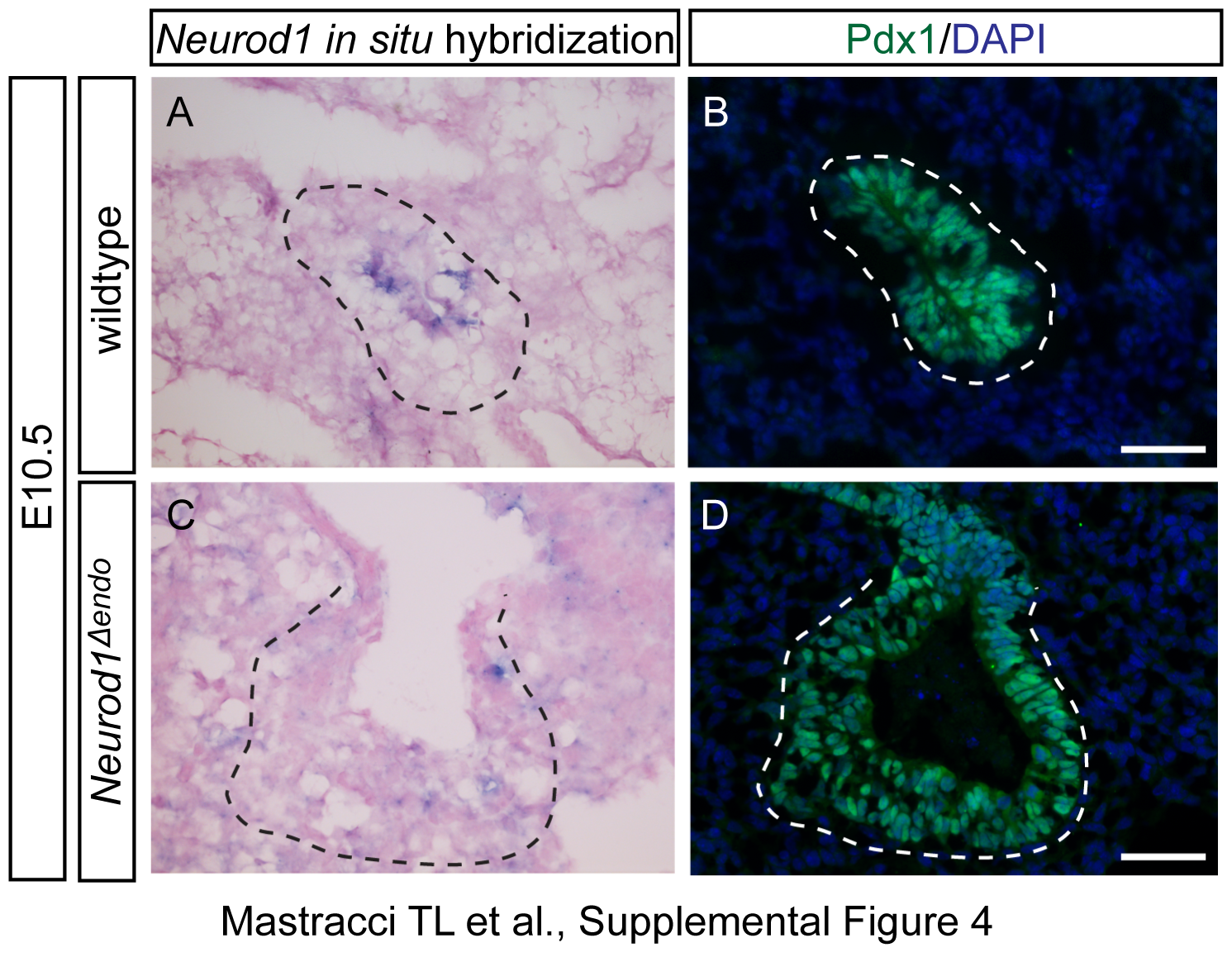

Supplement: Figure S4 — Neurod1 inactivation by Neurog3-cre in the early pancreatic bud. RNA in situ hybridization on pancreas sections from E10.5 wildtype (A) and Neurod1Δendo (C) embryos identified a reduction in Neurod1 by Neurog3-cre even at this early stage of development. Adjacent tissue sections were stained for Pdx1 (B, D) to identify the pancreas area (encircled with dashed lines). White bar indicates 50 microns. DAPI marks all nuclei. (TIF) [file pgen.1003278.s004.tif]

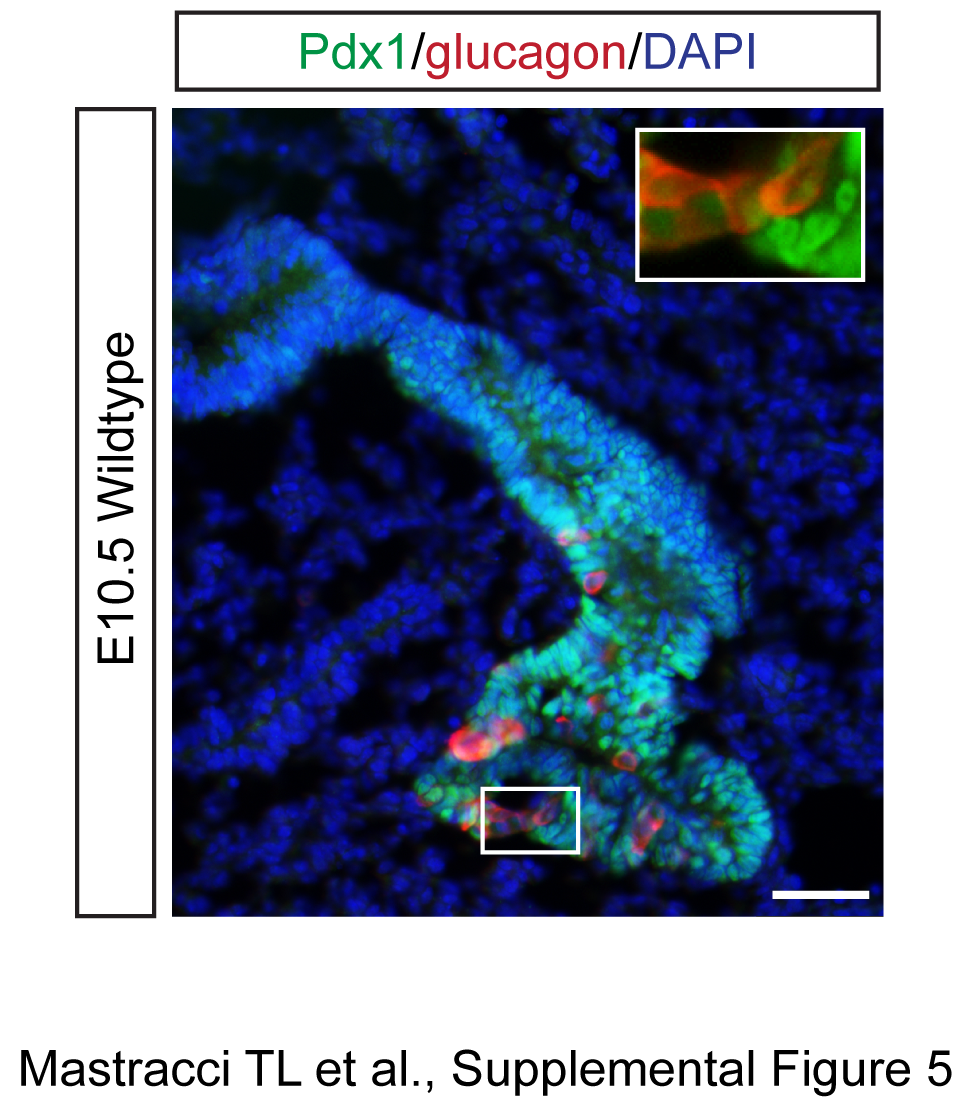

Supplement: Figure S5 — Alpha cells express low levels of Pdx1. A sagittal section through the dorsal pancreas of a wildtype E10.5 embryo was stained for Pdx1 and glucagon. Glucagon+ cells were observed to express low levels of Pdx1. Box denotes area magnified for inset but was imaged without DAPI; +1.75 zoom of low power image). White bar indicates 50 microns. DAPI marks all nuclei. (TIF) [file pgen.1003278.s005.tif]

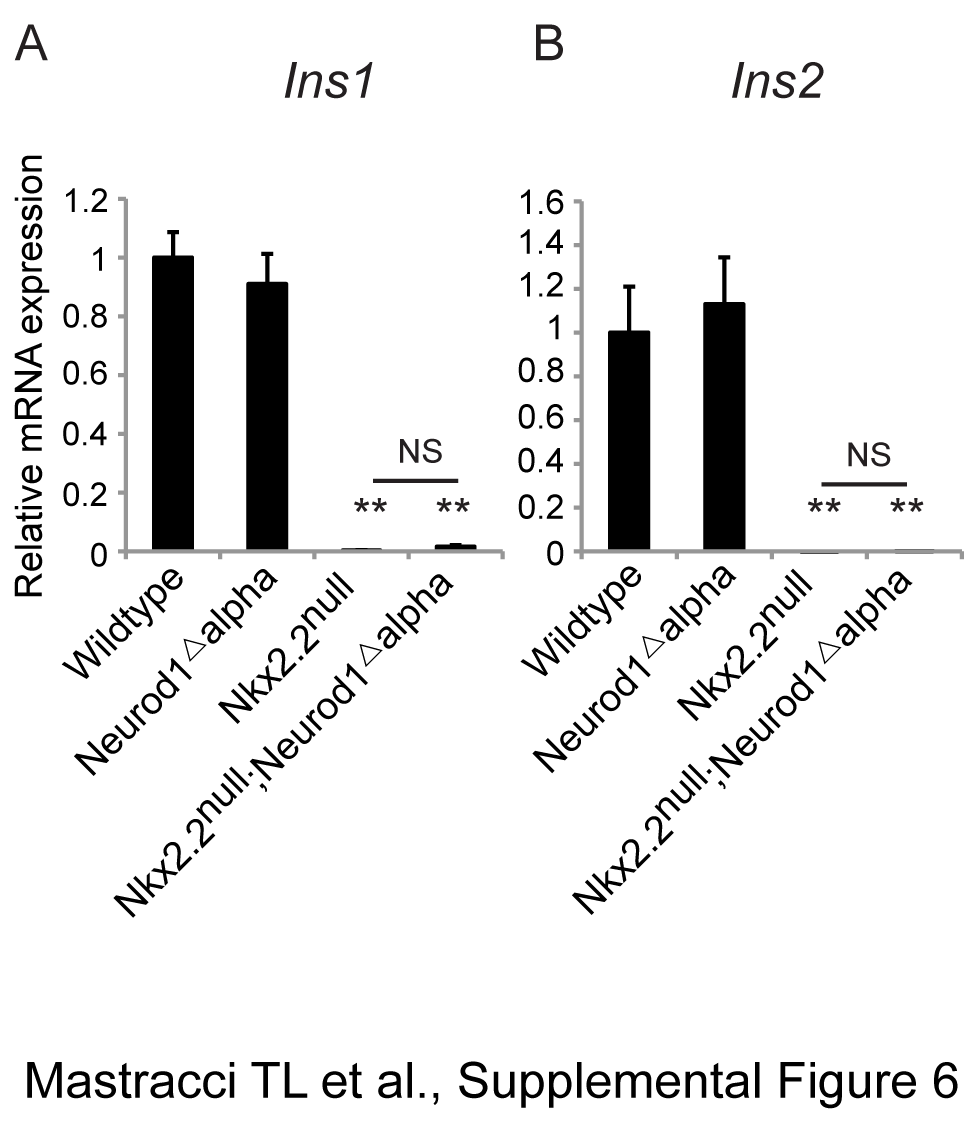

Supplement: Figure S6 — Insulin expression in the Nkx2.2null;Neurod1Δalpha. The quantitative expression of insulin1 (Ins1) (A) and insulin2 (Ins2) (B) was determined by real time PCR using RNA extracted from wildtype, Neurod1Δalpha, Nkx2.2null, and Nkx2.2null;Neurod1Δalpha pancreas (P0; N = 3–7). Relative mRNA expression was normalized to the housekeeping gene, cyclophilinB. Data are represented as mean+/−SEM. * p<0.05; ** p<0.01; *** p<0.001. (TIF) [file pgen.1003278.s006.tif]

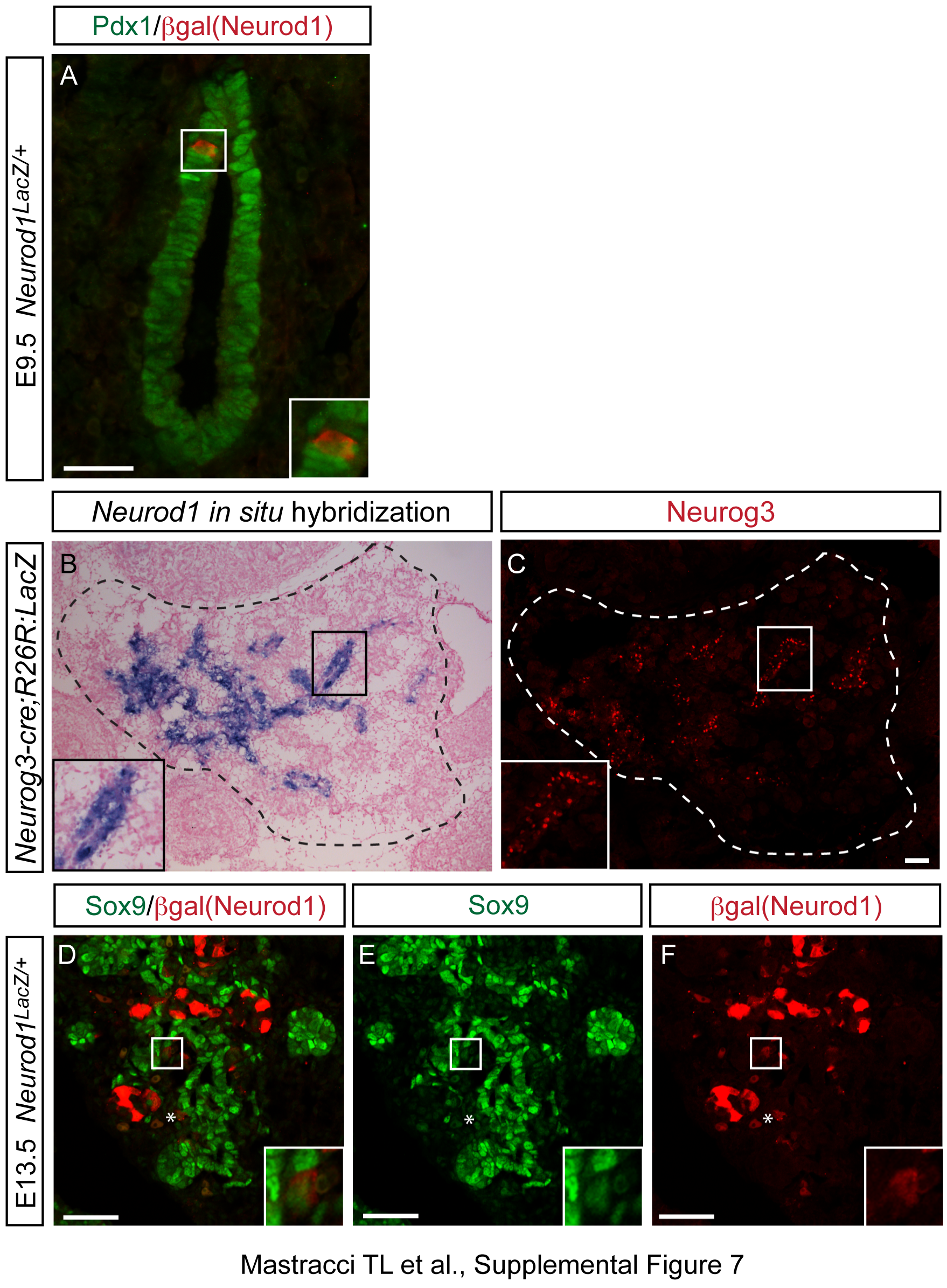

Supplement: Figure S7 — Neurod1 expression at specific developmental timepoints. Utilizing the Neurod1:LacZ knock-in allele (Neurod1LacZ/+) and immunofluorescence on tissues sections, Neurod1 (marked by beta-galactosidase; beta-gal) cells were identified to co-express Pdx1 at E9.5 (A). The overlap of Neurod1 and Neurog3 expression was identified at E15.5 by RNA in situ hybridization for Neurod1 (B) and immunofluorescent staining of Neurog3 (C) on the adjacent tissue section from a Neurog3-cre;R26R:LacZ embryo. A subset of Neurod1 cells that co-express Sox9 were also identified at E13.5 (D); the Sox9 (E) and beta-gal (F) channels were separated to visualize co-expressing cells more clearly. White bars indicate 50 microns. Boxes denote area magnified for inset, which are +1.75 zoom of lower power image. (TIF) [file pgen.1003278.s007.tif]
